# Genomic profiling of cefotaxime-resistant *Haemophilus influenzae* from Norway and Sweden reveals extensive expansion of virulent multidrug-resistant international clones

**DOI:** 10.3389/fmicb.2025.1601390

**Published:** 2025-07-29

**Authors:** Dagfinn Skaare, Inger Lill Anthonisen, Nermin Zecic, Andrew Jenkins, Dominique A. Caugant, Trond Egil Ranheim, Arnfinn Sundsfjord, Kristin Hegstad, Study Group on Exceptional Phenotypes in Haemophilus influenzae, Truls Michael Leegaard

**Affiliations:** Author Affiliations: (Akershus University Hospital, Lørenskog, Norway), (Innlandet Hospital, Lillehammer, Norway), (Haukeland University Hospital, Bergen, Norway), (Oslo University Hospital, Oslo, Norway), (St. Olav University Hospital, Trondheim, Norway), (Stavanger University Hospital, Stavanger, Norway), (Levanger Hospital, Levanger, Norway), (Vestre Viken Hospital Trust, Drammen, Norway), (Østfold Hospital, Grålum, Norway), (Førde Hospital, Førde, Norway), (Kalmar University Hospital, Kalmar, Sweden), (Sørlandet Hospital, Kristiansand, Norway), (Haugesund Hospital, Haugesund, Norway), (University Hospital of North Norway, Tromsø, Norway), (Møre og Romsdal Hospital Trust, Ålesund/Molde, Norway), (Sahlgrenska University Hospital, Gothenburg, Sweden), (Örebro University Hospital, Örebro, Sweden), (County Hospital Ryhov, Jönköping, Sweden), (Unilabs, Skövde, Sweden), (Nordland Hospital, Bodø, Norway).; ^1^Antimicrobial Resistance Research Group, Department of Microbiology, Vestfold Hospital Trust, Tønsberg, Norway; ^2^Department of Infection Prevention and Control, Vestfold Hospital Trust, Tønsberg, Norway; ^3^Department of Natural Science and Environmental Health, University of South-Eastern Norway, Bø, Norway; ^4^Department of Method Development and Analysis, Division for Infection Control, Norwegian Institute of Public Health, Oslo, Norway; ^5^Department of Community Medicine, Faculty of Medicine, University of Oslo, Oslo, Norway; ^6^Fürst Medical Laboratory, Oslo, Norway; ^7^Research Group for Host-Microbe Interactions, Department of Medical Biology, UiT – the Arctic University of Norway, Tromsø, Norway; ^8^Norwegian Centre for Detection of Antimicrobial Resistance, Department of Microbiology and Infection Control, University Hospital of North Norway, Tromsø, Norway; ^9^Centre for New Antibacterial Strategies, UiT – the Arctic University of Norway, Tromsø, Norway

**Keywords:** PBP3, *ftsI*, CRHI, BLNAR, BLPACR, cgMLST, LIN, cefotaxime

## Abstract

Cefotaxime-resistant *Haemophilus influenzae* (CRHI) are a global concern, but little is known about their molecular epidemiology. The goal of this study was to perform genomic profiling of 191 CRHI from Norway (*n* = 183) or Sweden (*n* = 8) (2006–2018) and assess clonal spread using core genome multilocus sequence typing (cgMLST)-based Life Identification Number (LIN) codes based on whole genome sequencing (Ion Torrent). Cefotaxime resistance was confirmed with broth microdilution minimal inhibitory concentration (MIC), interpreted with the European Committee on Antimicrobial Susceptibility Testing (EUCAST) breakpoints. 35.7% of isolates with cefotaxime gradient MIC of 0.25 mg/L were falsely resistant. All but two isolates (blood) were non-invasive, and all but two (serotype f) were non-typeable. Characterization included calling of resistance determinants, *ftsI* typing (penicillin-binding protein 3, PBP3), and classification of PBP3-mediated beta-lactam resistance (rPBP3), with assignment to rPBP3 stage and group. All isolates had rPBP3-defining substitutions, and 78.5% were stage 3 (L389F positive). Beta-lactam MICs correlated well with rPBP3 genotypes. Significant proportions of stage 3 isolates were cross-resistant to ceftriaxone (86.0%) and meropenem (meningitis breakpoints, 26.0%). The CRHI prevalence in Norway doubled during the study period and approached 1%. A shift from stage 2 to stage 3 rPBP3 in 2011–2012 led to emergence of CRHI with higher beta-lactam MICs and co-resistance to multiple non-beta-lactams, including extensively drug-resistant (XDR) strains. The shift was driven by transformation with two distinct variants of the transpeptidase region and multiclonal expansion. 66.0% of the isolates belonged to 27 clusters. Ten clusters or singletons belonged to international CRHI clones represented in the PubMLST database. The study provides new insight into CRHI evolution, resistance profiles, and clonal dynamics in a period when this phenotype went from exceptional to unusual in Europe. International CRHI clones are described for the first time, including eight high-risk clones associated with invasive disease, calling for enhanced genomic surveillance. LIN coding, supplemented with *ftsI* typing and rPBP3 staging, is well-suited for definition of CRHI clones. LIN9, defined by ≤ 10 allelic differences, offered the highest resolution level fully supported by maximum likelihood core genome phylogeny and is proposed as a global standard for genomic surveillance of *H. influenzae*.

## 1 Introduction

*Haemophilus influenzae*, a fastidious Gram-negative bacterium in the class Gammaproteobacteria, is a significant contributor to global morbidity, mortality, and antibiotic usage (Tristram et al., 2007; Nørskov-Lauritsen, 2014; Van Eldere et al., 2014; Slack et al., 2021)^1^. The organism has host specificity for humans and frequently colonizes the airways in all age groups (García-Rodríguez and Fresnadillo Martínez, 2002). Patients with chronic lung disease or immunosuppression, elderly people, pregnant women, young children, and neonates are at risk of severe infection, including bacteremic pneumonia and meningitis. While encapsulated strains of serotype b (Hib) are particularly virulent, strains of other serotypes and non-encapsulated strains, referred to as non-typeable (NTHi), cause the majority of invasive infections in the post-Hib vaccination era (Nørskov-Lauritsen, 2014; Van Eldere et al., 2014; Slack et al., 2021).

*H. influenzae* is naturally competent, i.e., able to exchange genetic material with other members of the species or closely related species such as *Haemophilus haemolyticus* and *Haemophilus parainfluenzae* by transformation (Takahata et al., 2007; Nørskov-Lauritsen, 2014; Witherden et al., 2014; Michel et al., 2024). Transformation facilitates the spread of gene variants giving advantageous phenotypes, such as antibiotic resistance (Takahata et al., 2007; Witherden et al., 2014; Hegstad et al., 2020; Michel et al., 2024). The process involves recognition and uptake of extracellular DNA, facilitated by the 9-bp uptake signal sequence (USS) ACCGCACTT in the donor molecule, followed by homologous recombination with the chromosome (Mell et al., 2012). Transformation is generally more frequent in NTHi than encapsulated strains, but recombination rates differ significantly between NTHi lineages (Meats et al., 2003; Connor et al., 2012; Nørskov-Lauritsen, 2014). Recombinational events have blurred but not eliminated the phylogenetic signal in *H. influenzae*, and the population structure is intermediate between clonal and panmictic, with two major divisions (Smith et al., 1993; Meats et al., 2003; Nørskov-Lauritsen, 2014). Whole genome-based phylogenetic approaches have identified distinct lineages with similar core and accessory genes (De Chiara et al., 2014; Lees et al., 2019).

Antibiotics are the mainstay in the management of severe *H. influenzae* infections. Dissemination of transferable beta-lactamase genes in the 1980s forced a shift from aminopenicillins to cephalosporins as first-choice empirical treatment (Tristram et al., 2007; Van Eldere et al., 2014). A new era started with the rapid emergence of cefotaxime-resistant *H. influenzae* in Japan around the millennium shift (Ubukata, 2003). The global AWARE study (2015–2016) pin-pointed Asia and to a certain degree Africa and the Middle East as hotspots for resistance to extended-spectrum cephalosporins (Bae and Stone, 2019). The phenotype has also emerged in Europe (García-Cobos et al., 2007; Skaare et al., 2014b; Van Eldere et al., 2014; Deghmane et al., 2019; Nürnberg et al., 2021). Comparison of cefotaxime and ceftriaxone resistance rates is hampered by different interpretive criteria recommended by the European Committee on Antimicrobial Susceptibility Testing (EUCAST)^2^ and the Clinical and Laboratory Standards Institute (CLSI)^3^. Resistance is highly infrequent by CLSI criteria because the minimal inhibitory concentration (MIC) breakpoints are four dilutions higher than corresponding EUCAST breakpoints. The best suited breakpoints to predict therapeutic success are debated in the absence of relevant clinical data (Merlino et al., 2023).

All known resistance to extended-spectrum cephalosporins in *H. influenzae* is caused by amino acid substitutions in penicillin-binding protein 3 (PBP3), an essential transpeptidase encoded by the *ftsI* gene, leading to impaired target affinity and antibacterial activity (Ubukata et al., 2001; Tristram et al., 2007; Bellini et al., 2019). PBP3 substitutions are acquired through spontaneous point mutations and/or transformation. Facilitated by naturally occurring USSs in the 5′ end of the transpeptidase region (nt 1006-1014) and 22 bp downstream of the *ftsI* gene, the entire gene may be transferred as a single recombinational event (Takahata et al., 2007; Witherden et al., 2014). The level of PBP3-mediated resistance (rPBP3) depends on the number of substitutions in key positions close to the conserved 512-Lys-Thr-Gly (KTG) and 379-Ser-Ser-Asn (SSN) motifs, which shape the active site pocket (Ubukata et al., 2001; Osaki et al., 2005; Tristram et al., 2007; Skaare et al., 2014b; Bellini et al., 2019).

The health burden and emerging beta-lactam resistance earned *H. influenzae* a spot on the World Health Organization (WHO) priority list to guide discovery, research and development of new antibiotics (see text footnote 1). The situation has been further complicated by the emergence of multidrug-resistant (MDR) and extensively drug-resistant (XDR) strains (Skaare et al., 2014b; Abavisani et al., 2024; Michel et al., 2024; Cherkaoui et al., 2025). Such profiles are associated with integrative conjugative elements (ICEs), self-transmissible mobile genetic elements capable of carrying resistance genes from genetically distant species (Juhas et al., 2007; Hegstad et al., 2020; Johannessen et al., 2022).

Clonal expansion of cefotaxime-resistant *H. influenzae* is well-described (Skaare et al., 2014b; Van Eldere et al., 2014). Earlier investigations have revealed both local outbreaks with suspected person-to-person transmission and national clusters with no obvious epidemiological links (García-Cobos et al., 2007; Skaare et al., 2014b; Nürnberg et al., 2021; Johannessen et al., 2022; Michel et al., 2024). However, for want of unambiguous, high-resolution typing methods suitable for comparison across investigator sites, little is known about the global molecular epidemiology. Whole genome sequencing represents a revolution in surveillance of antibiotic resistance in bacteria (Baker et al., 2023), and the recently published core genome multilocus sequence typing (cgMLST) scheme for *H. influenzae* provides a significantly improved framework for robust genomic tracking (Krisna et al., 2024). The protocol includes assignment of Life Identification Number (LIN) codes with 13 positions corresponding to thresholds from 0 to 910 allelic differences^4^, which provide a stable, multilevel taxonomy highly concordant with core genome phylogeny (Vinatzer et al., 2017).

The goal of this study was to perform genomic profiling of a historic collection of Norwegian and Swedish cefotaxime-resistant *H. influenzae* (CRHI), previously defined as an “exceptional phenotype” in Europe^5^, and assess domestic and cross-border clonal spread using LINs and the PubMLST genome database (Jolley et al., 2018)^6^.

## 2 Materials and methods

### 2.1 Selection and inclusion

*H. influenzae* with cefotaxime gradient MIC > 0.125 mg/L (2006-2018) were selected from (1) routine diagnostics at Vestfold Hospital Trust (VHT) (*n* = 34), (2) isolates with “exceptional phenotypes” (defined as resistance to any third-generation cephalosporin, carbapenems, or quinolones) submitted to VHT for confirmation and supplementary testing in line with EUCAST recommendations of the time (*n* = 222) (see text footnote 5), and (3) the Norwegian Surveillance System for Antimicrobial Drug Resistance (NORM) (*n* = 28)^7^ (Supplementary Figure S1).

Supplementary testing was performed according to routine diagnostic procedures at VHT. Species identification was done with conventional methods (factor X and V dependency and absence of beta-hemolysis on blood agar), and (from 2011) matrix-assisted laser desorption/ionization-time of flight (MALDI-TOF) mass spectrometry with MALDI Biotyper (Bruker Daltonics GmbH). Cefotaxime gradient MIC was determined with Etest (bioMérieux) (2006–2012) or MIC Test Strip (Liofilchem) (2012–2018), according to the manufacturers' recommendations. *H. influenzae* with confirmed cefotaxime gradient MIC > 0.125 mg/L (first isolate from each patient, n=213) were frozen at −70°C in Microbank vials (Pro-Lab Diagnostics, Ontario, Canada) pending further analyses.

Clinical data were collected from the referral forms and included sampling date, source, care level, age, sex, municipality and county of residence. Based on county of residence, Norwegian patients were assigned to six geographical regions, each comprising 10–25% of the population (East, 25%; West, 21%; South, 19%; Central, 14%; Capital, 12%; North, 10%) (Supplementary Figure S2)^8^. The regions corresponded to health regions, except that the South-Eastern region was split into Capital, East, and South. Sweden was counted as a single region.

### 2.2 Determination of resistance phenotypes

Broth microdilution (BMD) MIC was used as gold standard for evaluation of cefotaxime gradient tests, with calculation of full agreement, essential agreement (±1 dilution), and categorical agreement rates. Isolates not growing in the BMD assay (*n* = 2) and falsely cefotaxime-resistant isolates (gradient MIC > 0.125 mg/L and BMD MIC ≤ 0.125 mg/L) (*n* = 20) underwent sequencing to allow classification of penicillin-binding protein 3-mediated resistance (rPBP3) but were otherwise excluded from further analyses (Supplementary Figure S1).

BMD was done according to ISO 20776-1 on custom MIC panels (Sensititre NONAG7, Thermo Scientific), using Mueller-Hinton Fastidious (MH-F) broth (in-house produced with reagents from Oxoid), as recommended by EUCAST^9^. A total of 21 agents (nine beta-lactams and 12 non-beta-lactams) in 13 antimicrobial categories (five beta-lactams and eight non-beta-lactams) were tested (Supplementary Table S1).

Categorization as susceptible (S), susceptible, increased exposure (I), or resistant (R) was according to EUCAST clinical breakpoints (EUCAST v. 14.0, 2024) (see text footnote 2). 2024 breakpoints were used because BMD was performed with 0.06 mg/L as the lowest concentration for ciprofloxacin, corresponding to the epidemiological cut-off value (ECOFF) according to EUCAST prior to 2024^10^, which is incompatible with the breakpoint change from ≤ 0.06 mg/L to ≤ 0.03 mg/L in the most recent version of the breakpoint table (EUCAST v. 15.0, 2025). Resistance to ciprofloxacin may therefore be underestimated.

Before use in the study, NONAG7 was validated with MH-F broth (validated with *Haemophilus* Test Medium by the manufacturer). To cover calling ranges for as many agents as possible, a wide selection of reference strains was used for validation (*H. influenzae* ATCC 49247 and ATCC 49766, *Streptococcus pneumoniae* ATCC 40619, *Escherichia coli* ATCC 25922 and ATCC 35218, and *Pseudomonas aeruginosa* ATCC 27853). After validation, the quality control strains *H. influenzae* ATCC 49247 and ATCC 49766 were tested along with study isolates^11^.

Inoculation of MIC panels was done automatically (Sensititre AIM, Thermo Fisher Scientific). Plates were read and interpreted manually by one of the authors (DS), blinded for results from previous testing and molecular characterization, and photographed (Sensititre Vizion, Thermo Fisher Scientific) to allow reexamination and *post-hoc* comparison. Testing was done in duplicate and repeated in cases of discrepancy.

Classification of isolates as multidrug-resistant (MDR), extensively drug-resistant (XDR), or pandrug-resistant (PDR) was based on previously proposed principles (Magiorakos et al., 2012), except that “non-susceptible” was changed to “resistant” (MIC above the R breakpoint)^12^. Accordingly, MDR was defined as resistance to ≥1 agent in ≥3 antimicrobial categories, XDR was defined as resistance to ≥1 agent in all but one or two categories, and PDR was defined as resistance to all agents in all categories. Nine “epidemiologically significant” categories (five beta-lactams and four non-beta-lactams), defined as categories comprising agents with EUCAST breakpoints for therapeutic use, were counted (Supplementary Table S1).

### 2.3 Whole genome sequencing, phylogenetic analyses, and molecular typing

Frozen isolates were grown on chocolate agar for 16–20 h at 35°C with 5% CO_2_ and visually checked for purity. For each isolate, ~2.5 μl of colonial growth was transferred to a sterile tube with 500 μl of Tris-EDTA buffer and frozen at −70°C in Microbank vials. Genomic DNA was extracted from thawed bacterial suspension using QIAsymphony SP (QIAGEN, Germany). Whole genome sequencing was performed using Ion Torrent technology (Thermo Fisher Scientific). Library preparation was done with Ion Xpress Plus Fragment Library Kit for the AB Library Builder System, and the Ion Chef instrument was used for template preparation and loading of Ion Chips for sequencing with Ion S5XL. A physical version of the reference strain *H. influenzae* Rd KW20 (ATCC 51907) was included in the sequencing and bioinformatic procedures for quality control purposes.

Raw sequencing reads were quality-trimmed to Phred score ≥16. Samples with average read depth >30 underwent *de novo* assembly with Spades v.3.14.1^13^. Assembly files with <200 contigs and N50 >60 kb (Supplementary material) were annotated with Prokka v.1.14.6^14^. Output files from Spades and Prokka were used as inputs to an array of tools for molecular typing, phylogenetic analyses, and detection of transferable genes and chromosomal variants associated with antibiotic resistance (Supplementary Figure S3). Unlikely allele variants (e.g., frameshift mutations) were controlled manually with approaches based on reference-guided assembly of sequencing reads.

Capsular serotyping was done with hicap v. 1.0.3^15^. Multilocus sequence typing (MLST) (Meats et al., 2003) was done with mlst v.2.19.0^16^. Confirmed novel alleles and allelic profiles (STs) were submitted to the PubMLST database (Jolley et al., 2018) (see text footnote 6). PopPUNK v.2.7.0 (Lees et al., 2019) was used for assignment to distinct whole genome-based “strains” with a stable taxonomy, as defined in a database of reference genomes^17^.

Pangenome analysis was performed with Panaroo v.1.5.1, which uses an algorithm to correct for annotation errors suitable for highly recombinogenic bacteria (Tonkin-Hill et al., 2020)^18^. The core gene alignment file was used to construct a maximum likelihood (ML) phylogenetic tree with raxmlGUI v.2.0 (ML+ rapid bootstrap)^19^. The number of repeats (n=149) was determined with autoMRF. *H. haemolyticus* ATCC 33390 (GCA_004368535.1) was used as outgroup. Visualization of annotated phylogenetic trees and geo-mapping was done with Microreact v.261^20^.

Assembled genomes were submitted to PubMLST for cgMLST (1037 loci) (Krisna et al., 2024) (see text footnote 4). For isolates not reaching the threshold of ≤ 25 missing loci for assignment of Life Identification Number (LIN) codes, additional loci were called by reference-guided assembly with Minimap2 v.2.28^21^ and *H. influenzae* Rd KW20 (GCA_000027305.1) as reference. Life Identification Number (LIN) codes were used for assignment to clusters, defined by the LIN level offering the highest level of resolution fully supported by ML core genome phylogeny. Cluster analysis of isolates without LIN (>25 missing loci) was based on ML core genome phylogeny.

Finally, to assess the extent of cross-border clonal spread, we searched the PubMLST genome database for international clones, defined as groups of isolates from at least one country in addition to Norway and Sweden meeting the LIN-based cluster definition. Visualization of clusters of study isolates and international clones was done by construction of minimum spanning trees using the GrapeTree v.1.5.0 plugin^22^.

### 2.4 Antibiotic resistance determinants

Variants associated with resistance to beta-lactams (PBP3, HI_1132), quinolones (GyrA, HI_1264; ParC, HI_1529), or azithromycin (50S ribosomal proteins L4, HI_0778; L22, HI_0782) were called after multiple sequence alignment of translated DNA sequences using the msa R package v.1.24.0^23^, using *H. influenzae* Rd KW20 (GCA_000027305.1) as reference.

PBP3-mediated beta-lactam resistance (rPBP3) was classified according to a previously proposed three-stage system (Skaare et al., 2014b) with assignment to rPBP3 stage and group based on amino acid substitutions in key positions near the conserved 512-KTG and 379-SSN motifs in the transpeptidase region of PBP3, encoded by the *ftsI* gene (Table 1). The system is based on previous works (Ubukata et al., 2001; Osaki et al., 2005; García-Cobos et al., 2007) and was supplemented in the present study with the novel KTG-near first-stage substitutions N526H (Wienholtz et al., 2017) and Y528H (Thegerström et al., 2018).

**Table 1:** Classification of penicillin-binding protein 3-mediated beta-lactam resistance (rPBP3)^a.^

**Stage**	**Group**	**SSN-near substitutions**		**KTG-near substitutions** wicth 1pt			
		**S385T**	**L389F wicth 1pt**	**R517H**	**N526K**	**N526H^b^**	**Y528H^b^**
3	III(+)	+	+ wicth 1pt	–	+	–	–
	III-like(+)	+	+ wicth 1pt	+	–	–	–
2	III(-)	+	– wicth 1pt	–	+	–	–
	III-like(-)	+	– wicth 1pt	+	–	–	–
1	II	–	– wicth 1pt	–	+	–	–
	I	–	– wicth 1pt	+	–	–	–
	Misc1^b^	–	– wicth 1pt	–	–	–	+
	Misc2^b^	–	– wicth 1pt	–	–	+	–
0	None	–	– wicth 1pt	–	–	–	–

^*a*^Assignment to rPBP3 stage and group was based on the absence or presence of amino acid substitutions in key positions near the conserved 379-Ser-Ser-Asn (SSN) and 512-Lys-Thr-Gly (KTG) motifs in the transpeptidase region of PBP3, as proposed previously (Skaare et al., 2014b). The system is based on previous works (Ubukata et al., 2001; Osaki et al., 2005; García-Cobos et al., 2007) and was supplemented in the present study with the novel KTG-near first-stage substitutions N526H (Wienholtz et al., 2017) and Y528H (Thegerström et al., 2018).

^*b*^Novel substitutions or groups. Misc, miscellaneous.

Assignment to PBP3 types based on substitution patterns was done according to a previously established system, using letters for stage 1 and numbers for stage 2 and stage 3 rPBP3 (Skaare et al., 2014a,b). The fragment used for typing was extended from aa 338–573 to aa 327–610 in the present study to cover the complete transpeptidase region downstream of the active site Ser-327 (Bellini et al., 2019). Previously defined PBP3 types with variable substitution patterns in the extended fragments were divided into subtypes (e.g., 2a and 2b) to allow comparison with previous reports.

PBP3 substitutions in study isolates were aligned with the corresponding amino acid positions in the reference strains *H. haemolyticus* ATCC 33390 (WP_005643555.1) and *H. parainfluenzae* ATCC 33392 (WP_005696537.1).

The 855-bp fragment of the *ftsI* gene used for PBP3 typing (nt 979-1833) was used to construct a maximum likelihood phylogenetic tree with MAFFT v.1.4.0^24^ and raxmlGUI v.2.0 (ML+ rapid bootstrap) (see text footnote nineteen). The number of repeats (*n* = 600) was determined with autoMRF. Assignment to PubMLST *ftsI* “alleles” was based on the 621-bp fragment nt 976-1596 (erroneously described as nt 977-1597 in the protocol)^25^. Novel alleles were submitted to the PubMLST database (see text footnote six).

Classification of quinolone resistance mediated by alterations in the quinolone resistance-determining regions (QRDR) of GyrA and ParC (aa 80–92), with determination of QRDR stage (1–4), was based on amino acid substitutions in four key positions (aa 84 and 88 in both proteins) (Li et al., 2004).

Transferable resistance genes were called using ABRIcate v.1.0.1^26^ with the ResFinder^27^ and AMRFinderPlus^28^ databases (identity/coverage thresholds 75%). Hits <100% were investigated by BLASTN search^29^.

### 2.5 Software and statistics

Excel and PowerPoint 2016 (Microsoft Corporation, Redmond, WA) were used for handling and storage of de-identified data, creating diagrams, and editing of annotated phylogenetic trees. Statistical analyses were performed using SPSS Statistics version 26 (IBM Corporation, Armonk, NY) and Statistics Kingdom online calculators^30^, considering *p* < 0.05 statistically significant.

## 3 Results

### 3.1 The isolate collection was heterogeneous and nationwide

A total of 191 isolates of *H. influenzae* collected from different patients in Norway (*n* = 183) or Sweden (*n* = 8) between 2006 and 2018 were confirmed cefotaxime-resistant with reference methodology (BMD) and included in the study (Supplementary Figure S1). Subsets of isolates collected in the period 2006–2013 were characterized previously (Skaare et al., 2014b; Hegstad et al., 2020; Johannessen et al., 2022) (Supplementary material).

The isolates originated from 21 laboratories, 16 (of 18) Norwegian and five Swedish, and the patients resided in 14 (of 15) Norwegian and four (of 21) Swedish counties (Supplementary material). The annual number of isolates included was 1–5 from 2006 to 2012 and reached a maximum of 52 in 2017 (Supplementary Figure S2B). Regional distribution corresponded with regional population size, except that North was underrepresented, and Capital was overrepresented with isolates from primary care (Supplementary Figure S2C).

The patients were approximately equally distributed between primary care and hospital services (Supplementary Figure S4). Upper respiratory tract isolates predominated in primary care (87.9%), whereas a majority of hospital isolates (54.3%) were recovered from lower respiratory tract samples (including sputum and tracheal secretions). Two isolates were from blood cultures (1.0%).

Females were slightly overrepresented overall (*n* = 108, 56.5%), while sex distribution was close to equal among hospital patients (47/92 females, 51.8%). More than half of the patients were <10 yrs (22.5%) or ≥65 yrs (31.4%) of age. Median age was 62 yrs for hospital patients and 36 yrs for patients in primary care. Increasing age was a statistically significant independent risk factor for sampling from blood or lower respiratory tract (OR 1.26, 95% CI 1.16–1.36, *p* < 0.001) and hospital association (OR 1.11, 95% CI 1.05–1.18, *p* < 0.001) by multivariate (sex-adjusted) logistic regression analysis.

### 3.2 Gradient tests overcalled cefotaxime resistance

**Figure 1:**
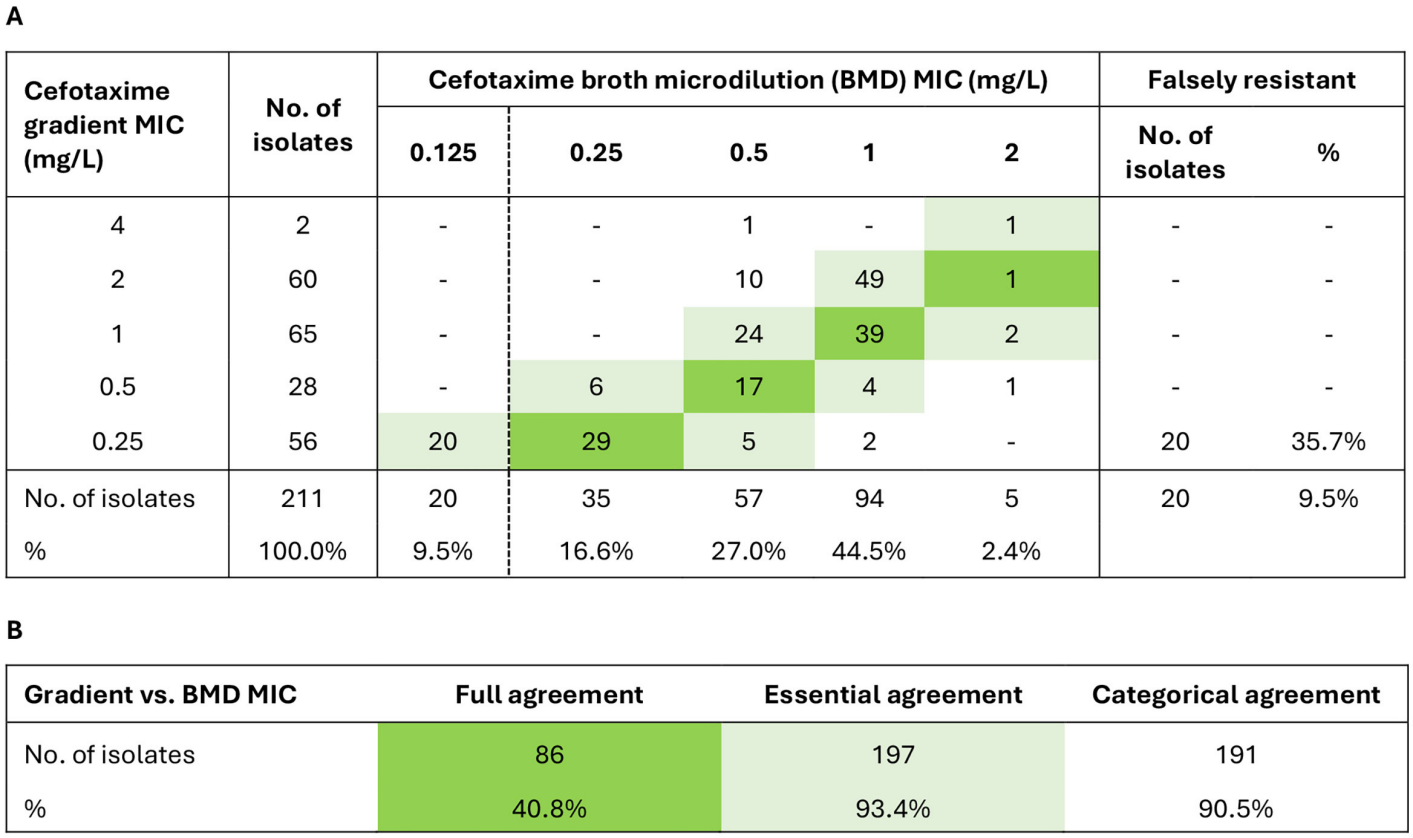
Evaluation of cefotaxime gradient MIC with broth microdilution (BMD) MIC as gold standard. The evaluation included 211 isolates with cefotaxime gradient MIC > 0.125 mg/L by supplementary testing (two isolates did not grow in the BMD MIC assay) (Supplementary Figure S1). **(A)** MIC correlations. **(B)** Agreement rates. Full agreement, identical MIC (green shades). Essential agreement, ±1 dilution (light green shades). Categorical agreement, identical categorization as susceptible (S) or resistant (R). The clinical breakpoint (S ≤ 0.125/R > 0.125 mg/L) is shown as a dashed vertical line in **(A)** (EUCAST v. 14.0, 2024) (see text footnote two).

Gradient tests overcalled cefotaxime MIC by ≥1 dilution in more than half (52.6%) of the isolates (Figure 1). Full, essential, and categorical agreement rates were 40.8%, 93.4%, and 90.5%, respectively. The false resistance rate was 9.5% overall, and 35.7% among isolates with gradient MIC just above the breakpoint (0.25 mg/L). Twenty isolates falsely resistant to cefotaxime by gradient MIC and two isolates not growing in the MIC assay (cefotaxime gradient MIC 0.25 mg/L and 0.5 mg/L) underwent sequencing to allow rPBP3 classification but were otherwise excluded from further analyses because the inclusion criterion of confirmed cefotaxime resistance was not met.

Twelve out of 28 isolates reported as cefotaxime-resistant to the Norwegian surveillance system (NORM) during the study period were excluded due to wrong species or cefotaxime gradient MIC ≤ 0.125 mg/L by supplementary testing (Supplementary Figure S1). Two surveillance isolates with cefotaxime gradient MIC of 0.25 mg/L were susceptible by BMD MIC (Supplementary Figure S5A), raising the overall false resistance rate to 50.0% in the NORM collection.

The CRHI prevalence among non-invasive isolates corresponded well with the annual number of isolates included (Supplementary Figure S3A). The prevalence increased from 0.4% to 0.7% between 2007 and 2017, with a further increase to 0.8% in 2022 as indicated by surveillance data (Supplementary Figure S5B). The overall CRHI prevalence among invasive isolates was 0.4% in 2012–2018, and more recent surveillance data suggest an increase to between 0.6% and 1.1% in 2019–2023 (Supplementary Figure S5C).

### 3.3 Cross-resistance and co-resistance were frequent and increasing

**Figure 2:**
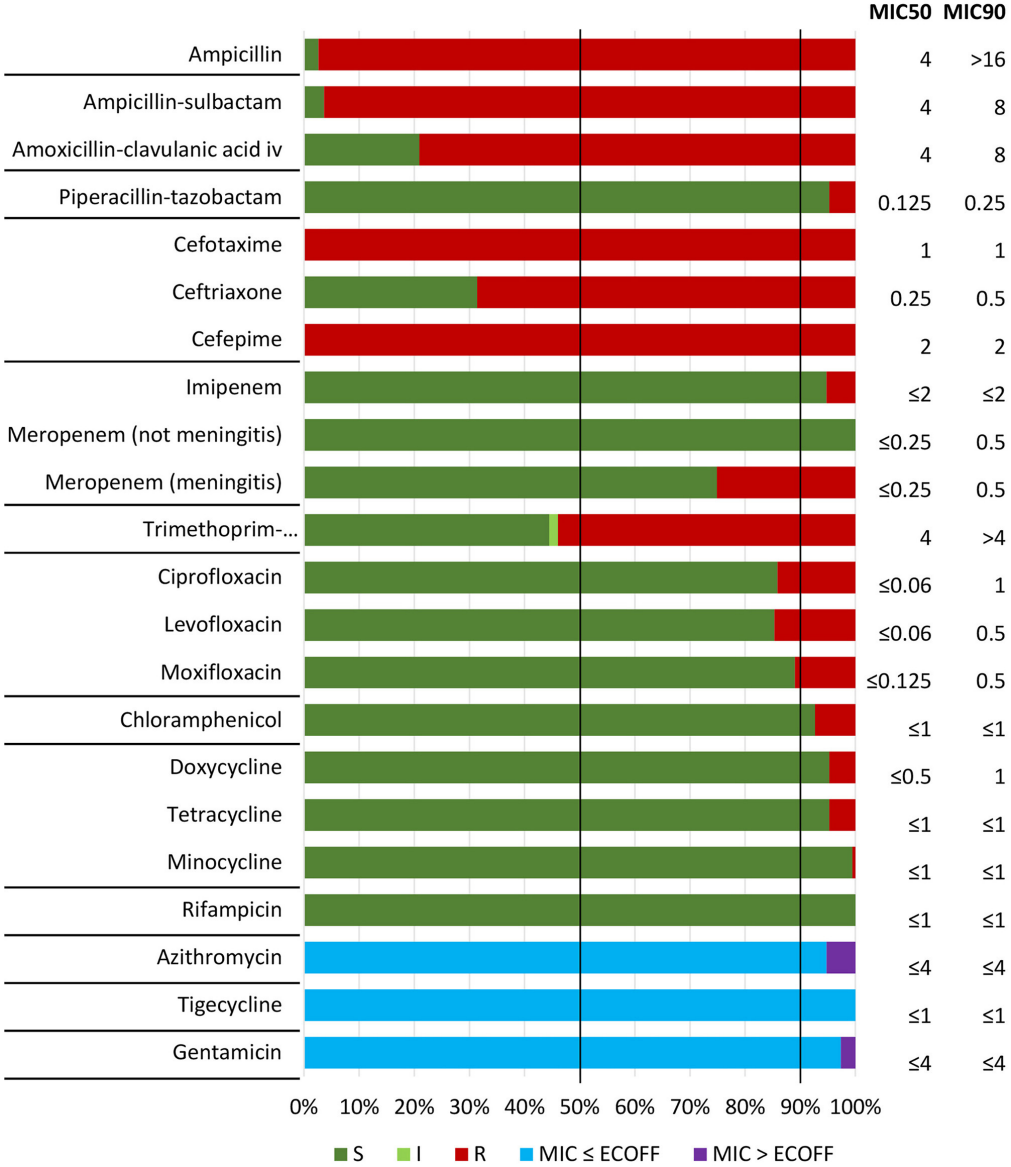
Antimicrobial susceptibility testing (broth microdilution MIC) of included isolates (*n* = 191) against nine beta-lactam and 12 non-beta-lactam antibiotics, with categorization as susceptible (S), susceptible increased exposure (I), or resistant (R) according to EUCAST clinical breakpoints (v. 14.0, 2024) (see text footnote two). Epidemiological cut-off values (ECOFFs) were used for assessment of susceptibility to agents without breakpoints (azithromycin, tigecycline, and gentamicin) (see text footnote ten). Horizontal lines separate antimicrobial categories (Supplementary Table S1). Vertical lines indicate MIC50 and MIC90, representing MIC values covering 50% and 90% of the isolates, respectively.

All 191 confirmed cefotaxime-resistant isolates (CRHI) were cross-resistant to cefepime, and 68.6% and 4.7% were cross-resistant to ceftriaxone and piperacillin-tazobactam, respectively (Figure 2). No CRHI were cross-resistant to meropenem by general breakpoints, but 48 (25.1%) were meropenem-resistant by meningitis breakpoints. Among non-beta-lactam antimicrobials, co-resistance was most frequent to trimethoprim-sulfamethoxazole (53.9%) and quinolones (14.7%). No isolates were resistant to rifampicin or tigecycline.

Phenotypic resistance profiles are shown in Supplementary Figure S6. Of particular note is that 29 CRHI (15.2%) were cross-resistant to both ceftriaxone and meropenem (meningitis breakpoints), including two (1.0%) with cross-resistance to piperacillin-tazobactam (Supplementary Figure S6A). More than half (56.5%) expressed resistance to at least one non-beta-lactam, and five (2.6%) were resistant to all four categories of non-beta-lactams with breakpoints for therapeutic use (Supplementary Figure S6C). Most CRHI (95.3%) were classified as multidrug-resistant (MDR), six (3.1%) were extensively drug-resistant (XDR), while none were pandrug-resistant (PDR) (Supplementary Figure S6D).

**Figure 3:**
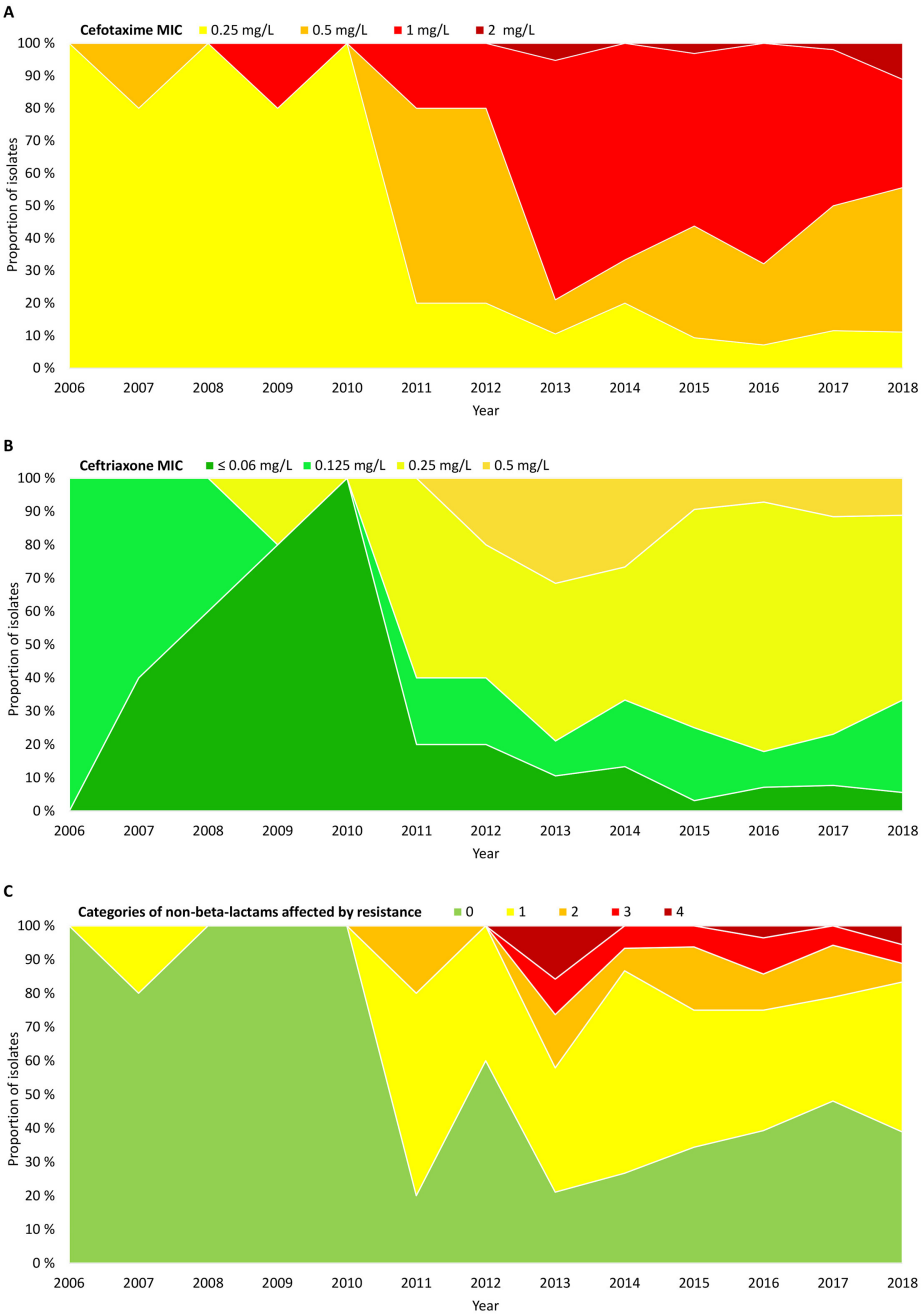
Timelines showing proportions of the included isolates (*n* = 191) by **(A)** cefotaxime MIC, **(B)** ceftriaxone MIC, and **(C)** extent of resistance to non-beta-lactams, expressed as the number of categories of non-beta-lactams affected by resistance (including categories without breakpoints) (Supplementary Table S1). Absolute numbers of isolates are shown in Supplementary Figure S2B.

A significant phenotypic shift occurred in 2011–2012, with two-dilution increases in median MICs for cefotaxime (from 0.25 mg/L to 1 mg/L) (Figure 3A) and ceftriaxone (from ≤ 0.06 mg/L to 0.25 mg/L) (Figure 3B). The shift led to emergence of CRHI with cross-resistance to ceftriaxone (Figure 3B) and co-resistance to ≥2 categories of non-beta-lactams (Figure 3C).

### 3.4 Core genome phylogeny and cluster analysis revealed a multiclonal population structure

**Figure 4:**
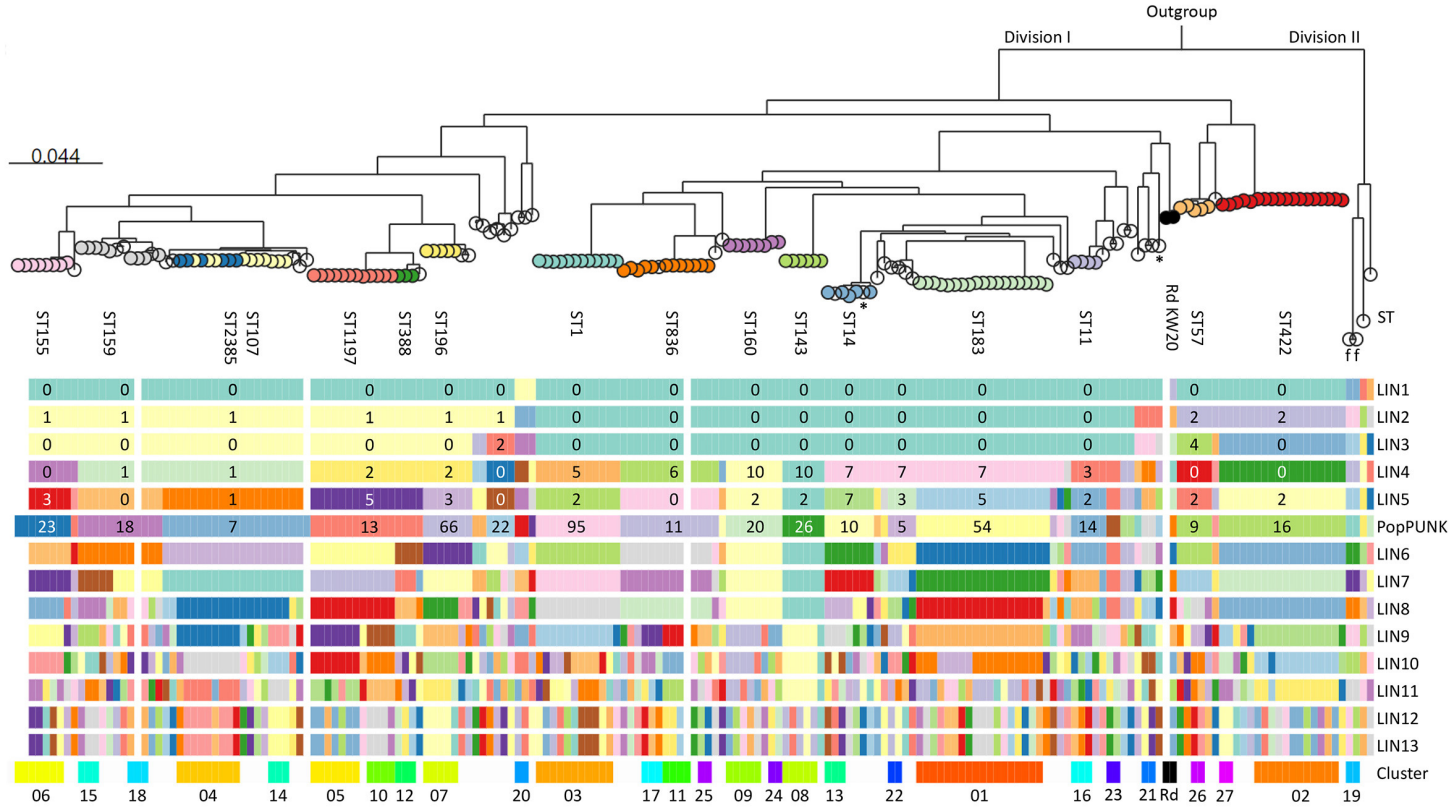
Maximum likelihood phylogenetic tree based on 1,286 core genes identified by pangenome analysis of the included isolates (*n* = 191) and *H. influenzae* Rd KW20 (ATCC 51907 and the reference sequence GCA_000027305.1). *H. haemolyticus* ATCC 33390 (GCA_004368535.1) was used as outgroup. Colored nodes, sequence types (ST) with ≥3 representatives (labeled). Transparent nodes, STs with 1–2 representatives. Black nodes, Rd KW20 (ST47 and ST1621). Invasive (*n* = 2) and encapsulated (*n* = 2) isolates are marked with asterisks and letters indicating serotype (f), respectively. Color bands show Life Identification Number (LIN) code (13 levels) derived from core genome multi-locus sequence typing (cgMLST) profiles (PubMLST protocol) (see text footnote four), PopPUNK strain (see text footnote seventeen), and clusters (01–27). PopPUNK strains and LIN5 with ≥3 representatives are labeled. Lacking LIN (five study isolates and the reference sequence) and no cluster assignment (singletons) are shown as transparent bands.

pangenome analysis identified 3,109 genes, of which 1,286 (41.4%) were core genes (present in ≥99% of the genomes). Median number of genes in each genome was 1,702 (range 1,613–1,928). The basal node of the maximum likelihood core genome phylogenetic tree (Figure 4) separated the population into two divisions. Division II contained only four isolates, including the only encapsulated isolates in the collection (serotype f, ST124, *n* = 2). Division I contained all the other isolates, all of which were non-typeable.

Fifty-four STs were represented, including five with novel combinations of known alleles (ST2341-ST2345), and two with novel alleles (ST2385, *frdB*-234; ST2386, *adk*-254). ST107 and the novel ST2385 intermingled by core genome phylogeny. The isolates belonged to 34 PopPUNK strains, four of which (comprising ST264, ST1221, ST1618, and ST2345) lacked in the reference database. Except for ST3, present in PopPUNK strains 5 and 21, all STs were confined to single PopPUNK strains.

All but five isolates (97.4%) passed the threshold of ≤ 25 missing loci and were assigned Life Identification Number (LIN) codes. Fifteen LINs (13 positions) were shared by 2–6 isolates, while 143 were present in single isolates. LIN5, corresponding to a threshold of 280 allelic differences, showed perfect correlation with “strain” assignment with PopPUNK. Meanwhile, LIN9, corresponding to a threshold of 10 allelic differences, offered the highest level of resolution fully supported by maximum likelihood core genome phylogeny and was used to define clusters.

Twenty-seven clusters of isolates with identical LIN9 were identified in 19 PopPUNK strains (Figure 4). Four isolates without LIN were assigned to clusters 06, 11, and 18 based on core genome phylogeny. Overall, 66.0% of the isolates belonged to a cluster, and the 11 largest (*n* = 4–18) accounted for 45.5% of the isolates (Supplementary Figure S7). Isolates with identical LIN9 had 0–23 allelic differences by minimum spanning analysis, whereas the four cluster members without LINs differed from the closest relative at 22–30 loci.

### 3.5 All isolates had rPBP3-defining substitutions, and stage 3 rPBP3 predominated

**Figure 5:**
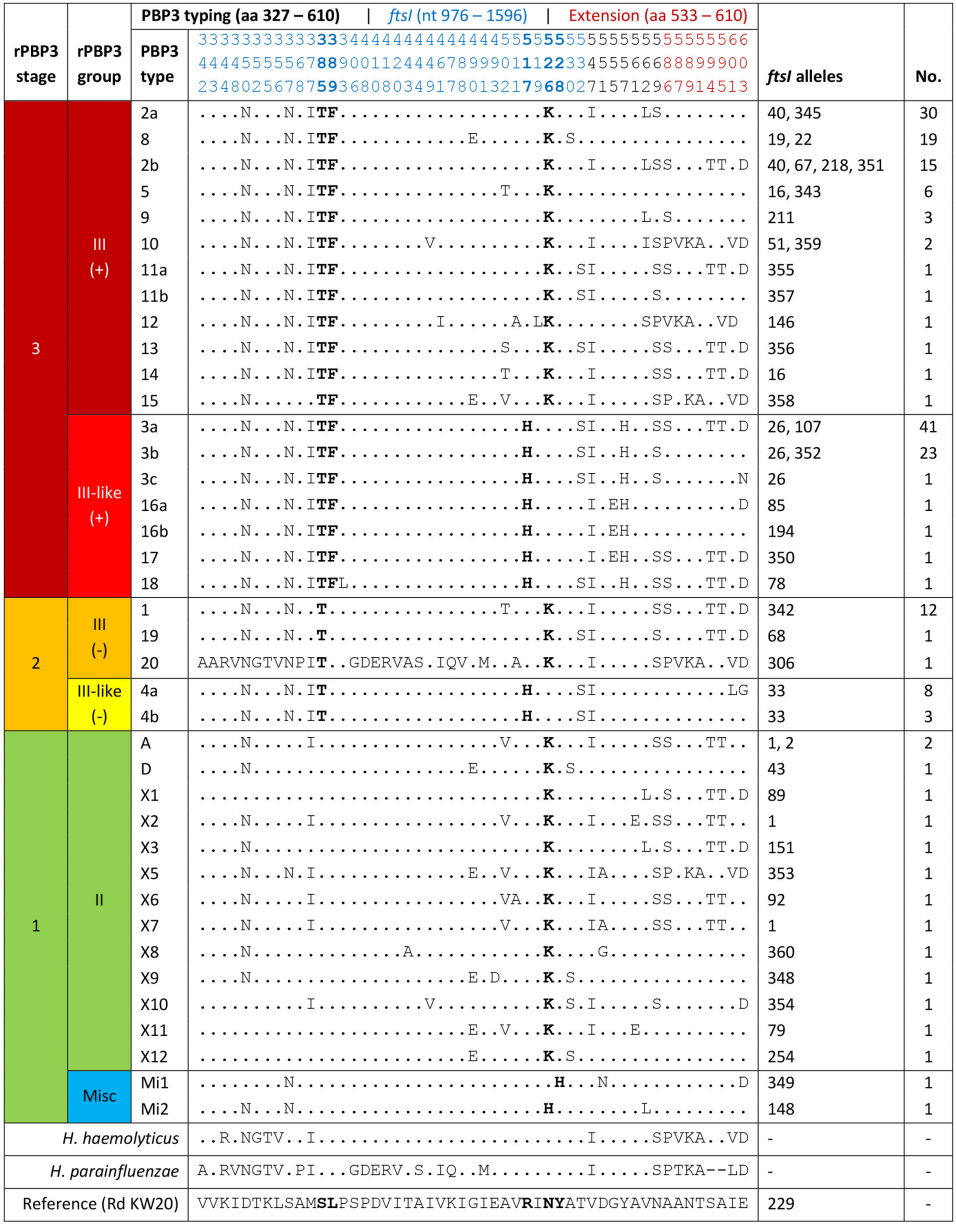
Classification of penicillin-binding protein 3-mediated resistance (rPBP3), with assignment of the included isolates (*n* = 191) to rPBP3 stage and group based on amino acid substitutions in key positions (bold) (Table 1). Assignment to PBP3 types based on substitution patterns was in line with a previously established system (letters for stage 1, numbers for stage 2–3) (Skaare et al., 2014a,b), except that the fragment was extended from aa 338–573 to aa 327–610. Previously defined PBP3 types with variable substitution patterns in the extended fragment (red positions) were divided into subtypes, e.g., 2a and 2b. Blue positions are encoded by the PubMLST *ftsI* allele (see text footnote twenty-five). For positions with observed substitutions (*n* = 51), the corresponding amino acids in the reference sequences of *H. haemolyticus* and *H. parainfluenzae* are shown. Dots, amino acids identical to *H. influenzae* Rd KW20 (reference). Misc, miscellaneous.

Analysis of translated *ftsI* sequences revealed amino acid substitutions in 51 positions in the transpeptidase region of PBP3 (aa 327–610) (Figure 5). In 26 positions, the substitution corresponded to the amino acid in the reference sequence of *H. haemolyticus* and/or *H. parainfluenzae*. All isolates possessed an rPBP3-defining first-stage substitution, in most cases N526K (57.1%) or R517H (41.9%). The majority (78.5%) also carried the second- and third-stage substitutions S385T and L389F and were classified as stage 3. The 20 excluded isolates falsely resistant by cefotaxime gradient MIC were rPBP3 stage 1 (*n* = 18) or 2 (*n* = 2), and the two isolates not growing in the BMD assay were stage 1 (Supplementary material).

Thirty-nine PBP3 types with 3-33 substitutions were detected, and the five most frequent were stage 3. Forty-two *ftsI* alleles were represented, 16 of which were novel (allele numbers between 342 and 360). Half (50.0%) of the stage 3 isolates carried one of the two most frequent alleles (*ftsI*-26 or *ftsI*-40). PBP3 types and *ftsI* alleles combined to 49 different PBP3-*ftsI* types, 15 of which accounted for 82.2%. The two most frequent (3a-26 and 2a-40) were carried by 33.5% of the isolates.

### 3.6 A shift to stage 3 rPBP3 was driven by multiclonal expansion and transformation

**Figure 6:**
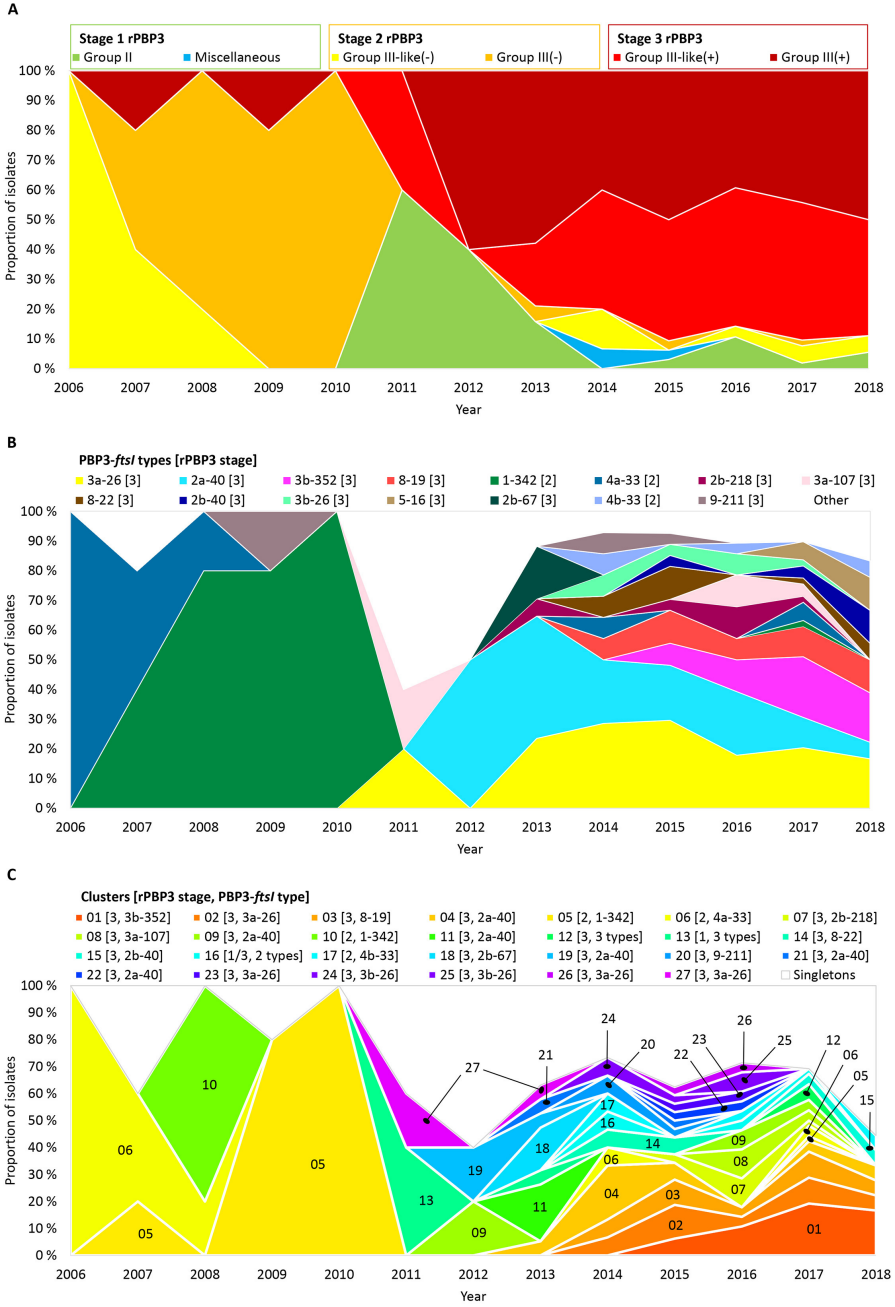
Timelines showing proportions of the included isolates (*n* = 191) by **(A)** rPBP3 stage and group, **(B)** PBP3-*ftsI* types (rPBP3 stage in brackets), and **(C)** clusters (same colors as in Figure 4 and Supplementary Figure S7). PBP3-*ftsI* types occurring in single isolates and isolates not belonging to clusters (singletons) are shown as white background in **(B)** and **(C)**. Absolute numbers of isolates are shown in Supplementary Figure S2B.

The phenotype shift in 2011–2012 (Figure 3) corresponded with a shift from stage 2 to stage 3 rPBP3 (Figure 6A). Before 2011, 88.2% (15/17) of CRHI were stage 2, while 85.1% (148/174) of CRHI sampled between 2011 and 2018 were stage 3. The shift was caused by the emergence of multiple novel PBP3-*ftsI* types, most of which encoded stage 3 (Figure 6B), driven by multiclonal expansion (Figure 6C). While most clusters emerging after the shift were stage 3, two early clusters with stage 2 types (clusters 05 and 06) persisted for more than 10 years.

**Figure 7:**
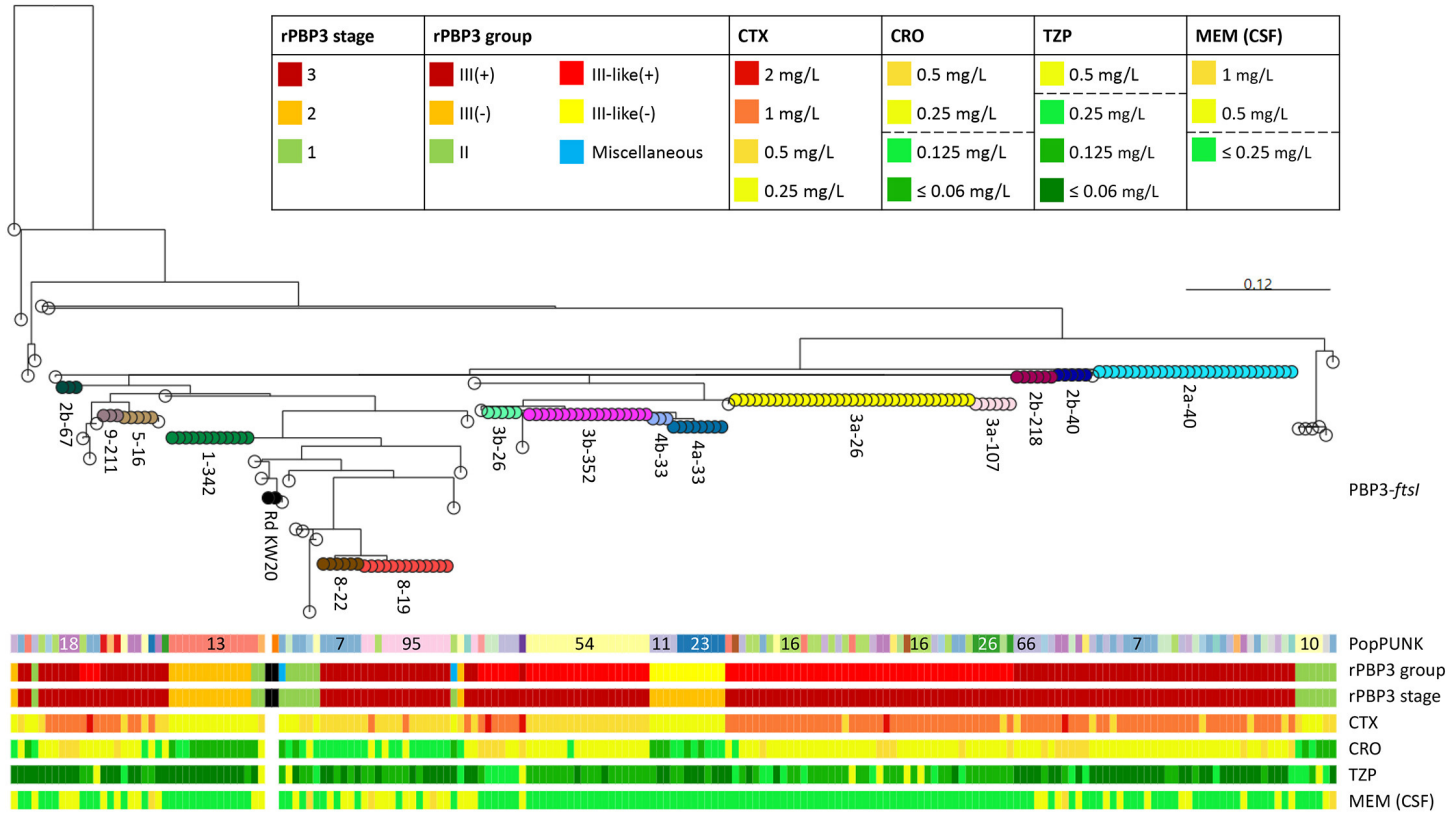
Maximum likelihood phylogenetic tree based on partial *ftsI* sequences (transpeptidase region, nt 979–1,833, aa 327–610) in the included isolates (*n* = 191) and *H. influenzae* Rd KW20 (ATCC 51907 and the reference sequence GCA_000027305.1). The most divergent sequence, encoding PBP3 type 20 (defined in Figure 5) was used as outgroup. Node colors and labels indicate combinations of PBP3 types and *ftsI* alleles (PubMLST protocol, nt 976–1,596) (see text footnote twenty-five), termed PBP3-*ftsI* types, present in ≥2 isolates (*n* = 15, range 3–35) (same colors as in Figure 6B and Supplementary Figure S8). Transparent nodes, PBP3-*ftsI* types present in single isolates (*n* = 34). Black nodes, Rd KW20. Color bands indicate PopPUNK strain (see text footnote seventeen), rPBP3 group and stage, and MICs for cefotaxime (CTX), ceftriaxone (CRO), piperacillin-tazobactam (TZP), and meropenem (MEM). MIC colors reflect categorization as susceptible or resistant according to EUCAST clinical breakpoints (legend, dashed lines) (see text footnote two). CSF, cerebrospinal fluid.

PBP3-*ftsI* type 3a-26 was present in six clusters and 14 singletons across 15 PopPUNK strains, while 2a-40 was present in six clusters and five singletons across 10 PopPUNK strains (Supplementary Figure S8). In both cases, the transpeptidase region was identical to DNA level in all isolates with identical PBP3-*ftsI* types, consistent with horizontal transfer by recombination (Figure 7). Isolates carrying either of the two variants had 1–2 dilutions higher cefotaxime MICs than isolates with other frequent PBP3-*ftsI* types.

### 3.7 Beta-lactam MICs corresponded with rPBP3 genotypes

Nearly all (99.3%) stage 3 isolates had cefotaxime MIC of 1 mg/L ±1 dilution, whereas 85.4% of stage 1 and stage 2 isolates had cefotaxime MIC of 0.25 mg/L (Supplementary Figure S9). A stage 3 genotype predicted cefotaxime MIC ≥0.5 mg/L with sensitivity, specificity, and positive and negative predictive values of 95.5%, 97.1%, 99.3%, and 82.9%, respectively. Most (86.0%) stage 3 isolates were resistant to ceftriaxone, whereas only 4.9% of stage 1 and stage 2 isolates were ceftriaxone-resistant.

While cefotaxime and ceftriaxone MIC generally corresponded well with rPBP3 stage, there were systematic differences between rPBP3 groups. Resistance to ceftriaxone was significantly more frequent among group III-like(+) isolates compared to group III(+) (97.1% vs. 76.5%, *p* < 0.001), mainly because isolates with PBP3 type 8 had one dilution lower ceftriaxone (and cefotaxime) MIC than other group III(+) isolates (Figure 7).

Group III-like(+) isolates had higher median piperacillin-tazobactam MIC compared to group III(+), and a significantly higher proportion of the isolates had MIC above the breakpoint (7.8% vs. 1.3%, *p* = 0.050) (Supplementary Figure S9). Conversely, no group III-like(+) isolates and almost half (48.1%) of group III(+) isolates were resistant to meropenem in cases of meningitis (*p* < 0.001). Although group III(+) isolates comprised 81.3% of isolates with meropenem MIC above the meningitis breakpoint, isolates with stage 1 rPBP3 types were associated with higher resistance rates (43.8%) than stage 2 (8.0%) and stage 3 (26.0%).

Cefotaxime MIC differed consistently between stage 3 isolates with PBP3 type 3b, apparently depending on *ftsI* allele (*ftsI*-26 or *ftsI*-352). However, the two variants occurred in different PopPUNK strains (Figure 7).

### 3.8 CRHI were significantly more often resistant to non-beta-lactams

**Figure 8:**
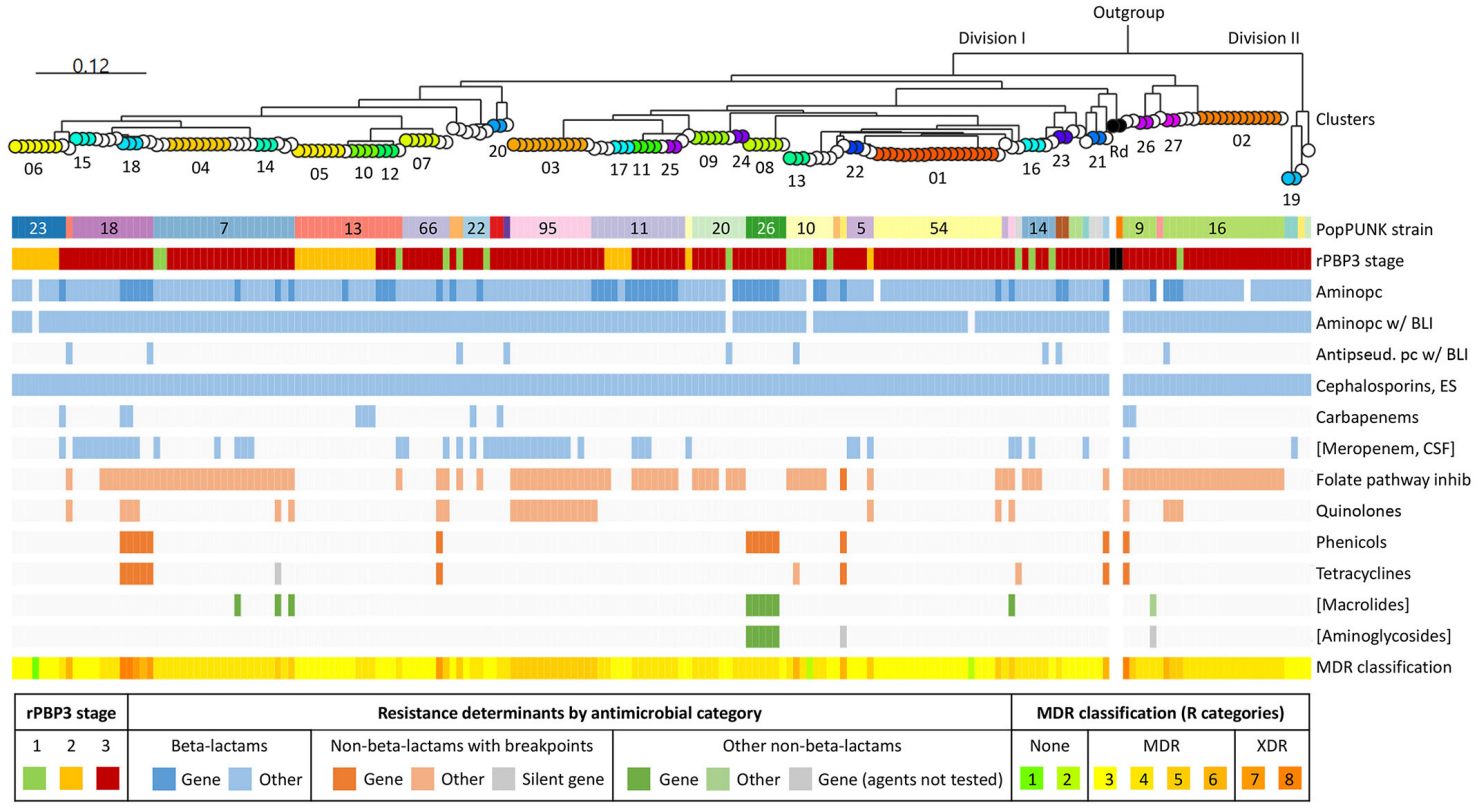
Core genome phylogeny and antibiotic resistance. Maximum likelihood phylogenetic tree based on 1,286 core genes identified by pangenome analysis of the included isolates (*n* = 191) and *H. influenzae* Rd KW20 (ATCC 51907 and the reference sequence GCA_000027305.1) (same tree as in Figure 4). *H. haemolyticus* ATCC 33390 (GCA_004368535.1) was used as outgroup. Node colors and labels indicate clusters (01–27) (same colors as in Figures 4, 6C, and Supplementary Figure S7). Singletons have transparent nodes. Black nodes, Rd KW20. Color bands show PopPUNK strain (see text footnote seventeen), rPBP3 stage, and antibiotic resistance profiles. Blue, beta-lactams; red, “epidemiologically significant” non-beta-lactams (breakpoints for therapeutic use); green, other non-beta-lactams (Supplementary Table S1). Darker colors indicate acquired antibiotic resistance genes. Gray, silent gene. Classification of multidrug resistance was based on nine antimicrobial categories (disregarding agents and categories in brackets). Pc, penicillin; BLI, beta-lactamase inhibitor; ES, extended-spectrum; CSF, cerebrospinal fluid; MDR, multidrug-resistant; XDR, extensively drug-resistant.

Figure 8 shows an overview of the population structure and antibiotic resistance profiles. Four of six XDR isolates, including the three in cluster 18, were ST159 and belonged to PopPUNK strain 18. The other two were ST57 and ST1521 (PopPUNK strains 9 and 66, respectively). Five of six XDR were resistant to trimethoprim-sulfamethoxazole, quinolones, chloramphenicol, and tetracyclines.

Forty-eight isolates (25.1%) carried acquired antibiotic resistance genes encoding penicillinase (*bla*_TEM − 1B_ or *bla*_TEM − 1C_) or resistance to chloramphenicol (*cat*A2), tetracyclines [*tet*(B) or *tet*(M)], sulfonamides (*sul*2), macrolides (*mef* (E) and *mel*), gentamicin (*aph*(2^′′^)-Ib and *aac*(6′)-Im), streptomycin (*strA* and *strB*), or kanamycin (*aph*(3′)-Ia) (Supplementary Figure S10). The most frequent was *bla*_TEM − 1B_ (24.6%).

Nine different profiles of acquired resistance genes were observed, and solitary *bla*_TEM − 1B_ (14.7%) was the most frequent. Seven profiles, present in 19 (9.9%) stage 3 rPBP3 isolates, contained non-beta-lactam resistance genes. The 19 isolates included cluster 18 (profile 2) and cluster 08 (profile 3). Previous in-depth characterization of representatives for the two clusters confirmed that all resistance genes were carried on the integrative conjugative elements (ICE) Tn*6686* (Hegstad et al., 2020) and Tn*7100* (Johannessen et al., 2022), respectively.

Acquired resistance genes caused most cases of resistance to chloramphenicol, tetracyclines, azithromycin, and gentamicin (Supplementary Figure S11). On the other hand, only one incidence of trimethoprim-sulfamethoxazole resistance and none of quinolone resistance could be attributed to known acquired resistance genes. All quinolone-resistant isolates had alterations in QRDR of GyrA and/or ParC, and MICs increased with the number of substitutions. Eight of nine isolates carrying *tet*(B) were susceptible to minocycline. One isolate carried a silent *tet*(M) gene with a deletion (nt 668) causing a premature stop codon. One azithromycin-resistant isolate without known acquired macrolide resistance genes had L4 and L22 substitutions that were absent in all other isolates.

Resistance to trimethoprim-sulfamethoxazole, ciprofloxacin, chloramphenicol, and tetracycline was significantly more frequent among CRHI (all agents *p* < 0.001), and stage 3 rPBP3 had the highest resistance rates (Supplementary Figure S12). Stage 3 isolates were also resistant to a wider range of non-beta-lactams than other isolates, and all six XDR isolates were stage 3 (Supplementary Figure S13).

### 3.9 Extensive domestic and international spread of CRHI clones

The 11 largest clusters were disseminated to from one to four (of seven) geographical regions (Supplementary Figure S14). Clusters 03 and 25 comprised both Norwegian and Swedish isolates (Supplementary material).

Direct import was suspected for seven (7.6%) of 92 clones (27 clusters and 65 singletons). These included two XDR isolates associated with recent travels to the Canary Islands or several countries in Southeast Asia, the first isolates in clusters 10 (Egypt) and 12 (Cuba), and three singletons (Japan, Vietnam, and the Canary Islands).

To further investigate international dissemination, we searched the PubMLST database for CRHI with LIN9 identical to study isolates (Nov 29, 2024, 6.552 genomes). Limiting the search to LIN9 associated with stage 3 PBP3 types (22 clusters and 48 singletons), we found matching database genomes for 15 of 70 (21.4%). Nine of these 15 international clones were “pure” CRHI clones entirely composed of CRHI with identical stage 3 rPBP3-encoding *ftsI* alleles, while six were mixtures of non-CRHI and CRHI. One of six mixed clones comprised an international CRHI subclone, while five mixed clones did not comprise CRHI other than study isolates.

**Figure 9:**
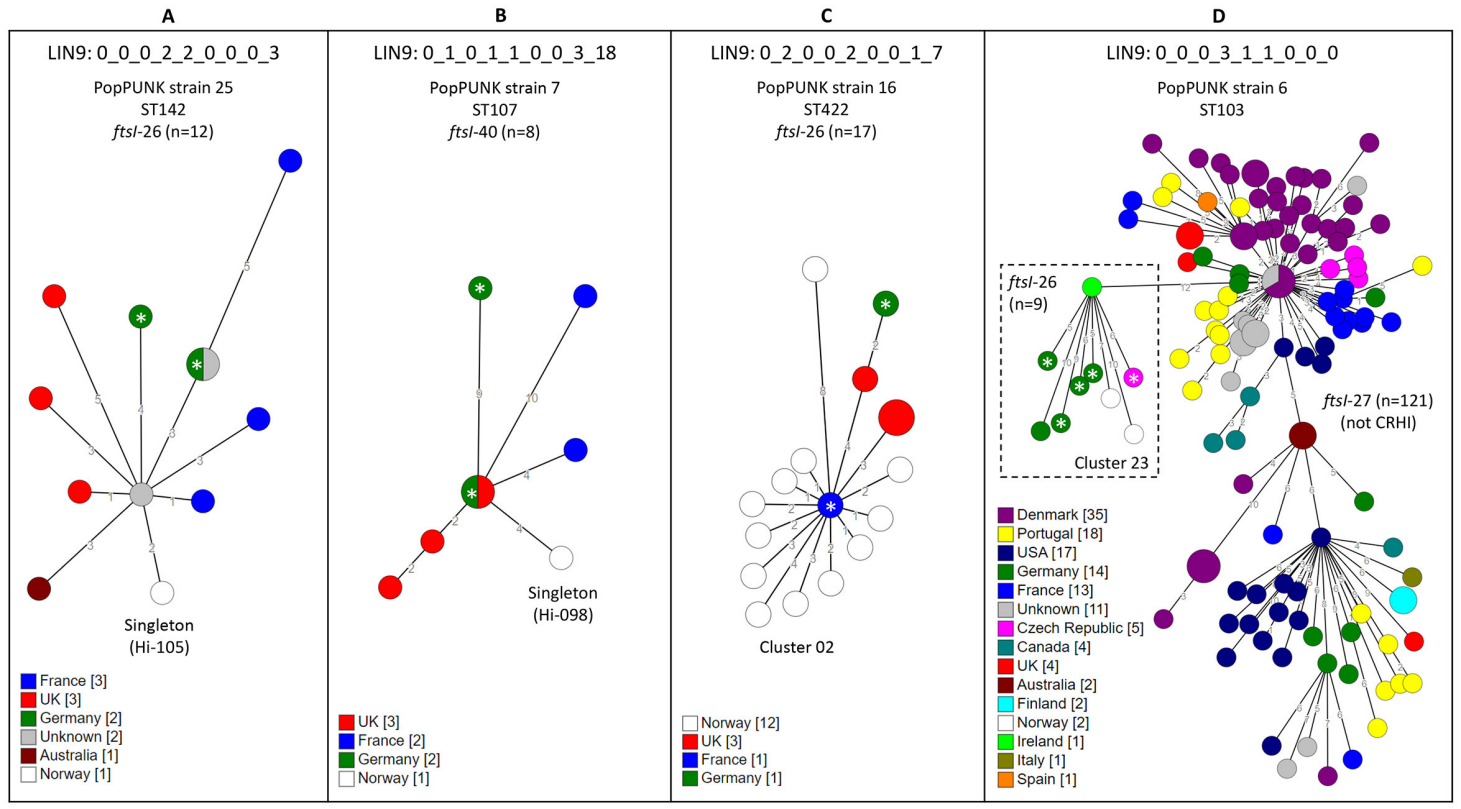
Minimum spanning trees (MST) of clones of cefotaxime-resistant *H. influenzae* (CRHI) with members from three or more countries in addition to study isolates. The clones were identified through a search in the PubMLST database (2024-11-29, 6.552 genomes) (see text footnote six) for isolates with Life Identification Number (LIN) code corresponding to a threshold of ≤ 10 allelic differences (LIN9) identical to clusters or singletons in the present study. The search was limited to clones with stage 3 rPBP3 types because of the strong association with cefotaxime resistance. **(A–C)** show clones consisting entirely of CRHI isolates, while **(D)** shows a CRHI subclone carrying *ftsI*-26 (dashed box) emerging from a non-CRHI clone carrying *ftsI*-27, which does not encode rPBP3-defining substitutions. The trees were calculated from cgMLST profiles (PubMLST protocol, 1,037 loci) (see text footnote four) using the GrapeTree plugin (see text footnote twenty-two). Node colors by country. Numbers along lines connecting nodes indicate allelic differences by MST analysis. Larger nodes comprise two or more isolates with identical cgMLST profiles. Invasive CRHI are marked with asterisks.

Overall, we identified 10 international CRHI clones or subclones with members from the present study. Three pure CRHI clones (one comprising cluster 02 and two comprising singletons) and the CRHI subclone (comprising cluster 23) had members from at least three countries in addition to Norway and Sweden, and one CRHI clone had members from two continents (Figure 9). The remaining six CRHI clones (comprising clusters 03, 08, and 15, and three singletons) (Supplementary material) encompassed isolates from one or two European countries (France, Germany, and/or UK). Seven of the ten international CRHI clones or subclones carried *ftsI*-26 (*n* = 5) or *ftsI*-40 (*n* = 2), and eight comprised invasive isolates.

## 4 Discussion

Extended-spectrum cephalosporins, such as cefotaxime, are important for empirical treatment of severe infections, making the global emergence of cefotaxime-resistant *H. influenzae* (CRHI) a cause for growing concern (Van Eldere et al., 2014). To the best of our knowledge, the current study represents the largest and most comprehensive genomic investigation of CRHI to date. It is also the first to apply the recently introduced cgMLST-based Life Identification Number (LIN) code system to the epidemiology of CRHI. The study provides new insight into the evolution, clonal dynamics, and resistance mechanisms of CRHI in a period when this phenotype went from exceptional to unusual in Europe (see text footnote five).

Our results and recent surveillance data suggest that the CRHI prevalence in Norway doubled during the study period and is now approaching 1%. This is comparable to Germany, where 0.9% of invasive *H. influenzae* were cefotaxime-resistant in 2016–2019 (Nürnberg et al., 2021). A significant shift took place in Norway in 2011–2012, with the emergence of CRHI with higher cefotaxime MIC, higher rates of cross-resistance to ceftriaxone and meropenem (in meningitis), and higher rates of co-resistance to multiple non-beta-lactams. The shift was driven by multiclonal, cross-border expansion and horizontal transfer of two distinct *ftsI* fragments comprising the entire transpeptidase region of penicillin-binding protein 3 (PBP3).

In this study, as previously, we favor the term “rPBP3” to denote PBP3-mediated beta-lactam resistance (Skaare et al., 2014a). The term is independent of concomitant beta-lactamase production, does not exclude important phenotypes (such as CRHI), and is not associated with phenotype-genotype conflicts, in contrast to the widely used multicomponent constructs “gBLNAR” (genetically beta-lactamase negative ampicillin-resistant) and “gBLPACR” (genetically beta-lactamase positive amoxicillin-clavulanic acid-resistant). Using a previously proposed three-stage classification system reflecting the stepwise development and gradually increasing resistance levels of rPBP3 strains (Skaare et al., 2014b), expanded with the novel first-stage substitutions N526H and Y528H in the present study (Wienholtz et al., 2017; Thegerström et al., 2018; Bellini et al., 2019), we found that all CRHI had rPBP3-defining substitutions, confirming that PBP3 alterations thus far remain paramount for cefotaxime resistance in *H. influenzae*.

Cefotaxime MIC generally corresponded well with rPBP3 stage, confirming the previously reported strong correlation between MIC and *ftsI* alleles (Taha et al., 2022). A stage 3 genotype predicted cefotaxime MIC > 0.25 mg/L (one dilution higher than the current EUCAST breakpoint) with high sensitivity (95.5%) and specificity (97.1%), suggesting that rPBP3 staging has the potential for sequence-based susceptibility categorization. Systematic discrete differences between stage 3 rPBP3 groups were noted for some beta-lactams, reflecting that resistance levels to a certain degree depend on which first-stage substitution is present (Osaki et al., 2005; Skaare et al., 2014a). Notably, while N526K usually confers higher beta-lactam MICs than R517H, the latter was associated with higher MICs for piperacillin-tazobactam.

It is plausible that PBP3 substitutions other than those included in the current version of the classification system may impact resistance levels. Based on transformation and site-directed mutagenesis experiments, the authors of a Japanese investigation concluded that the G555E and Y557H substitutions caused exceptionally high cefotaxime MICs (8–16 mg/L) in stage 3 group III-like(+) isolates (Mizoguchi and Hitomi, 2019). This, however, was not confirmed in the present study, where three group III-like(+) isolates with G555E and Y557H had cefotaxime MICs of 1–2 mg/L. A possible explanation for the discrepancy is that our isolates lacked the rare or previously unreported SSN-near T371I and KTG-near I519V substitutions, both present in all isolates and transformants with cefotaxime MIC ≥ 8 mg/L in the Japanese study. Additional investigations are required to clarify whether acquisition of T371 and/or I519V may further increase cefotaxime MIC in stage 3 rPBP3 carrying G555E and Y557H.

On the other hand, group III(+) isolates with PBP3 type 8 had consistently one dilution lower cefotaxime and ceftriaxone MICs than other group III(+) isolates. PBP3 type 8 contains the G490E and A530S substitutions, frequently present in stage 1 types but rare in stage 2 and stage 3 types, but it is unknown whether these substitutions impact the phenotype. Other notable observations were that cefotaxime MIC consistently differed between distantly related groups of stage 3 isolates with identical PBP3 types, and that a handful of stage 1 isolates had higher-than-expected cefotaxime MICs (0.5 mg/L). These observations suggest that genetic background may modify PBP3-mediated beta-lactam resistance. A recent genome-wide association study (GWAS) identified several genes associated with ampicillin resistance, including *ompP2* (Diricks et al., 2024), supporting early reports that outer membrane proteins may modulate beta-lactam MICs in *H. influenzae* (Regelink et al., 1999), but similar investigations focusing of cefotaxime resistance have to our knowledge not been performed.

Reliable methods for phenotypic susceptibility testing are crucial for adequate antibiotic therapy and representative surveillance data. Accurate tests are particularly important to avoid categorical errors when the test population is divided by the clinical breakpoint (Skaare et al., 2015). We found that stage 1 and stage 2 isolates, including 20 excluded, had cefotaxime MICs on both sides of the breakpoint, and gradient tests frequently caused major errors. More than one third (35.7%) of isolates with cefotaxime gradient MIC just above the clinical breakpoint (0.25 mg/L) were susceptible by BMD in the present study. The false resistance rate for isolates with cefotaxime gradient MIC of 0.25 mg/L was even higher (57.1%) in a German study (Nürnberg et al., 2021). These observations highlight the importance of confirming cefotaxime resistance with reference methodology. As an example, gradient tests suggested that 5.9% of invasive *H. influenzae* were cefotaxime-resistant in a French study, but the true CRHI prevalence was likely lower as three of eight isolates reported as resistant had gradient MIC of 0.25 mg/L (Deghmane et al., 2019).

Reference analyses of CRHI should include high-resolution epidemiological typing, and whole genome sequencing seems ideal for this purpose. Standardizing protocols and harmonizing interpretive criteria is essential for implementation of international genomic surveillance systems (Baker et al., 2023). We used cgMLST-derived Life Identification Number (LIN) codes to define clusters and assess the extent of clonal expansion (Vinatzer et al., 2017; Krisna et al., 2024). An important advantage of LIN-based analyses compared to the traditional minimum spanning approach is that addition of new isolates does not lead to merging or splitting of groups (Vinatzer et al., 2017). Furthermore, the multilevel taxonomy allows for tailoring the resolution level to the specific purpose. Importantly, LIN5, corresponding to a threshold of 280 allelic differences, showed perfect correlation with whole genome-based PopPUNK strains, representing distinct groups of isolates with similar core and accessory genomes (Lees et al., 2019). Accordingly, LIN5 may be suitable for defining clades and studying associations between population structure, virulence determinants, and disease (De Chiara et al., 2014; Vinatzer et al., 2017). A higher resolution level is required for surveillance of clones of interest. However, because genetic diversity inevitably increases with each generation, an epidemiologically meaningful LIN-based clone definition should represent the optimal balance between discriminatory power and sensitivity, and it is generally accepted that “clone” may be applied to closely related multidrug-resistant bacteria even in the absence of obvious epidemiologic connections (Woodford et al., 2011). LIN9, corresponding to a threshold of 10 allelic differences, offered the highest resolution level fully supported by maximum likelihood core genome phylogeny and was therefore used for cluster definition in the present study. The high discriminatory power of LIN9 was illustrated by clusters 05 and 10, previously interpreted as a single clonal group (“CG2”) based on pulsed-field gel electrophoresis band patterns (Skaare et al., 2014b). While genetic relationship between the two clusters indeed was confirmed by identical LIN8, corresponding to a threshold of 40 allelic differences, separation was supported by geo-mapping. Furthermore, the LIN9-based approach was robust over time, with more than 10 years separating the first and last member of individual clusters. Based on performance in the present study, we propose LIN9 as a global genomic standard for definition and surveillance of *H. influenzae* clones of interest.

The CRHI population was multiclonal, with 66.0% of isolates belonging to 27 clusters, 11 of which accounted for almost half (45.5%) of the isolates in the collection. Ten internationally distributed CRHI clones represented in the PubMLST genome database were represented by clusters or singletons in our collection. These shared identical LIN9 and identical stage 3-encoding *ftsI* alleles with the database entries (Jolley et al., 2018). To the best of our knowledge, these ten clones represent the first well-documented examples of cross-border expansion of CRHI. The strong European predominance among the matching database entries could result from database bias rather than true geographic restriction, as high-prevalent continents such as Asia and Africa (Bae and Stone, 2019) are underrepresented in the database (see text footnote six), limiting conclusions about global dissemination. Notably, eight international CRHI clones encompassed invasive isolates, fitting the description of high-risk clones (Woodford et al., 2011). Apart from warranting enhanced surveillance (Van Eldere et al., 2014), this illustrates that invasive and non-invasive *H. influenzae* often belong to the same clone, with invasive CRHI merely representing tips of multiple CRHI icebergs. Surveillance of CRHI should therefore include both invasive and non-invasive isolates to avoid underestimation of clonality (Van Eldere et al., 2014). As an example, the five largest international CRHI clones identified in this study comprised isolates from a genomic investigation of invasive *H. influenzae* in Germany, undermining the authors' conclusion that “rarely occurring cefotaxime resistance is caused by sporadic mutations” (Nürnberg et al., 2021).

One cluster in the present study (cluster 23) belonged to an international CRHI clone which appears to be a subclone derived from a *bla*_TEM_-positive ST103 clone associated with invasive infection (Tønnessen et al., 2022). The original clone, which dates back to 1995, carries *ftsI*-27 and lacks rPBP3-defining substitutions^31^, whereas the CRHI subclone has acquired the stage 3 rPBP3-encoding *ftsI*-26 and consists of more recent isolates (2015–2023) (see text footnote six). This 621-bp allele, which encodes ~73% of PBP3 type 3a, was by far the most frequent in the present study, and also the most frequent among non-invasive CRHI in a French study (2017–2021) (Taha et al., 2022). Identical copies of a larger, 855-bp fragment encoding PBP3-*ftsI* type 3a-26, representing the most frequent variant of the complete transpeptidase region in the present study, were present in multiple clusters and singletons across 15 PopPUNK strains. The same applied to the second most frequent type, 2a-40, strongly suggesting that transformation involving the two fragments has occurred repeatedly, leading to emergence of several novel CRHI clones. Both variants conferred higher cefotaxime MICs than less frequent PBP3-*ftsI* types, which might have contributed to their remarkable success. A selection advantage, fueled by a 200% increase in cefotaxime usage in Norway between 2000 and 2012 (see text footnote seven), is also a plausible explanation for the 2011–2012 shift from stage 2 to stage 3 rPBP3. Although far less dramatic, the shift mimicked the rapid emergence of stage 3 CRHI in Japan in the 1990s, attributed to massive use of oral cephalosporins (Ubukata, 2003). Notably, cefotaxime resistance in *H. influenzae* may also be induced by aminopenicillins (Jakubu et al., 2024), and the consumption of oral amoxicillin is almost 10 times higher than cefotaxime in Norway, with a 75% increase between 2000 and 2012 (see text footnote seven).

While cross-resistance to ceftriaxone was frequent, most CRHI were susceptible to piperacillin-tazobactam and meropenem, suggesting these last-resort beta-lactams are safe empirical therapy in severe CRHI infections. Meningitis is a notable exception, as every fourth isolate overall and almost half (48.1%) of stage 3 group III(+) isolates were resistant to meropenem by the breakpoints recommended by EUCAST in cases of meningitis (R > 0.25 mg/L). This finding suggests that empirical treatment with meropenem in CRHI meningitis may be associated with a significant risk of therapy failure. It should be noted that most isolates were susceptible to meropenem according to CLSI recommendations (S ≤ 0.5 mg/L) (see text footnote three). Piperacillin-tazobactam is currently not recommended for treatment of meningitis, but experiences from Japan and pharmacokinetic and pharmacodynamic (PK/PD) calculations suggest that the drug could be a valuable supplement for treatment of meningitis caused by *H. influenzae* with reduced susceptibility to first-line agents (Fukasawa et al., 2013).

Oral treatment options in less severe CRHI infections were restricted by significantly increased resistance rates to trimethoprim-sulfamethoxazole, tetracyclines, and quinolones (*p* < 0.001), and resistance to tetracycline and ciprofloxacin was more than 10 times higher among CRHI (see text footnote seven). The extent of multidrug resistance among CRHI was assessed using previously proposed criteria, adapted to *H. influenzae* in accordance with the logic of the original publication (Magiorakos et al., 2012). We defined agents with clinical breakpoints for therapeutic use as “epidemiologically significant” and included agents from all categories. The term “non-susceptible” was interpreted as MIC above the R breakpoint, in line with redefinition of the intermediate category to “susceptible, increased exposure” (see text footnote twelve). With these stringent criteria, we identified six (3.1%) extensively drug-resistant (XDR) isolates, all collected after the 2011–2012 genotype shift, reflecting that the shift also implied emergence of CRHI with higher rates of co-resistance to non-beta-lactams. A likely explanation for these observations is that extensive use of macrolides and tetracyclines due to the coinciding *Mycoplasma pneumoniae* epidemic (Blix et al., 2015) enhanced selection of multidrug-resistant (MDR) strains and mutator strains with defects in DNA repair or error avoidance systems, and boosted acquisition of a wide range of antibiotic resistance determinants through transformation and horizontal gene transfer (Blázquez et al., 2012). Two XDR isolates were recovered from patients with recent travels to Southeast Asia or the Canary Islands, reflecting that import of CRHI from regions with higher resistance rates also contributed to the high rates of co-resistance in our collection (Abavisani et al., 2024).

All XDR and most other isolates resistant to tetracyclines, chloramphenicol, azithromycin, or gentamicin carried known acquired resistance genes, and all isolates with acquired non-beta-lactam resistance genes were stage 3 rPBP3. Previous in-depth characterization of clusters 18 and 08 showed that all acquired resistance genes were located on the ICEs Tn*6686* and Tn*7100*, respectively (Hegstad et al., 2020; Johannessen et al., 2022). Cluster 18 (aka “CG4” or “strain G”) comprised three XDR ST159 isolates collected during a hospital outbreak in 2013 (Skaare et al., 2014b; Hegstad et al., 2020). The cluster belonged to PopPUNK strain 18 (LIN5 0_1_0_1_0), which also comprised a fourth XDR ST159 isolate. Notably, an XDR ST159 stage 3 group III(+) CRHI was recently reported from Switzerland (Cherkaoui et al., 2025). The resistance profile resembled that of cluster 18, except that the Swiss isolate was resistant to azithromycin and susceptible to chloramphenicol. Furthermore, while cluster 18 carried PBP3 type 2b, the Swiss isolate had a substitution pattern corresponding to PBP3 type 8 with one additional substitution (A586S). These observations illustrate the impressive capability of the genetic lineage LIN5 0_1_0_1_0 to acquire both chromosomal and transferable resistance determinants.

Worryingly, *bla*_CTX − M−15_ genes encoding extended-spectrum beta-lactamase (ESBL) were recently reported in *H. parainfluenzae* with cefotaxime MIC > 16 mg/L in Spain (Saiz-Escobedo et al., 2023) and France (Caméléna et al., 2024). The genes were located on Tn*3*-like transposons inserted into ICEs with backbones similar to Tn*6686* and Tn*7100* (Hegstad et al., 2020; Johannessen et al., 2022). As transposons and ICEs are shared between *H. influenzae* and *H. parainfluenzae* (Juhas et al., 2007; Carrera-Salinas et al., 2023), emergence of ESBL in *H. influenzae* in the near future is a likely scenario. The situation calls for enhanced surveillance and thorough characterization of CRHI with alarming phenotypes (Van Eldere et al., 2014; Baker et al., 2023). As an example, an invasive *H. influenzae* with cefotaxime and meropenem gradient MICs > 16 mg/L was recently reported from Belgium, but the phenotype was not confirmed with reference methodology, and the resistance mechanism was not investigated beyond *ftsI* sequencing (El Nouwar et al., 2025).

This study has some weaknesses. Because routine test panels differ between laboratories and submission of isolates with exceptional phenotypes was optional, the collection of non-invasive isolates is likely not complete. However, the number of annually included isolates corresponded well with adjusted surveillance data, supporting that the collection was representative for the CRHI epidemiology in Norway during the study period. Another weakness is that isolates collected later than 2018 were not included. This was compensated for by comparison with more recent genomes in the PubMLST database (see text footnote six). Furthermore, Ion Torrent sequencing technology has known weaknesses which may affect assembly quality, particularly for repetitive regions. To minimize artifacts, we combined *de novo* assembly with reference-guided approaches and used a pangenome analysis tool (Panaroo) which corrects for annotation errors (Tonkin-Hill et al., 2020).

To conclude, we provide the first evidence of international CRHI clones, including high-risk clones associated with invasive disease. A significant shift took place in Norway in 2011–2012, with emergence of CRHI with higher cefotaxime MICs, cross-resistance to ceftriaxone and meropenem (in meningitis), and co-resistance to multiple non-beta-lactams, including XDR strains. The shift was mainly driven by transformation with two distinct *ftsI* variants and multiclonal, cross-border expansion, fueled by increased selective antibiotic pressure. The finding of extensive clonal expansion is an important reminder of the formidable capacity of *H. influenzae* for person-to-person transmission and the importance of adequate infection prevention and control precautions in health institutions. The results also underline the importance of rational antibiotic usage and call for enhanced genomic surveillance, which should include both invasive and non-invasive CRHI. LIN coding, supplemented with *ftsI* typing and rPBP3 staging, is well-suited for definition and recognition of CRHI clones. LIN9, defined by ≤ 10 allelic differences by cgMLST, offered the highest resolution level fully supported by maximum likelihood core genome phylogeny and is proposed as a global standard for genomic surveillance of *H. influenzae*.

## Study Group on Exceptional Phenotypes in *Haemophilus influenzae*

Truls Michael Leegaard (Akershus University Hospital, Lørenskog, Norway), Tine Smedsund Dons (Innlandet Hospital, Lillehammer, Norway), Paul Christoffer Lindemann (Haukeland University Hospital, Bergen, Norway), Karianne Wiger Gammelsrud (Oslo University Hospital, Oslo, Norway), Kjersti Wik Larssen (St. Olav University Hospital, Trondheim, Norway), Iren Høyland Löhr (Stavanger University Hospital, Stavanger, Norway), Kyriakos Zaragkoulias (Levanger Hospital, Levanger, Norway), Roar Magne Bævre-jensen (Vestre Viken Hospital Trust, Drammen, Norway), Sara Debes (Østfold Hospital, Grålum, Norway), Reidar Hjetland (Førde Hospital, Førde, Norway), Annika Carlsson Wistedt (Kalmar University Hospital, Kalmar, Sweden), Ståle Tofteland (Sørlandet Hospital, Kristiansand, Norway), Liv Jorunn Hafne (Haugesund Hospital, Haugesund, Norway), Gunnar Skov Simonsen (University Hospital of North Norway, Tromsø, Norway), Einar Nilsen (Møre og Romsdal Hospital Trust, Ålesund/Molde, Norway), Brynja Ármannsdóttir (Sahlgrenska University Hospital, Gothenburg, Sweden), Martin Sundqvist (Örebro University Hospital, Örebro, Sweden), Anna Åkerlund (County Hospital Ryhov, Jönköping, Sweden), Carina Thilesen (Unilabs, Skövde, Sweden), Sandra Åsheim (Nordland Hospital, Bodø, Norway).

## Data Availability

Nucleotide sequences from this study have been deposited in the European Nucleotide Archive (ENA) at the European Molecular Biology Laboratory (EMBL-EBI) under BioProject accession number PRJEB49398 (individual accessions listed in Supplementary material)^32^.

## Footnotes

^1^WHO: Priority pathogens list. Accessed Feb 21, 2025. https://www.who.int/publications/i/item/WHO-EMP-IAU-2017.12.

^2^EUCAST: Clinical breakpoints. Accessed Feb 21, 2025. https://www.eucast.org/clinical_breakpoints.

^3^CLSI: M100. Accessed Feb 21, 2025. https://clsi.org/standards/products/microbiology/documents/m100/.

^4^PubMLST: Scheme information – cgMLST v1. Accessed Feb 21, 2025. https://pubmlst.org/bigsdb?db=pubmlst_hinfluenzae_seqdef&page=schemeInfo&scheme_id=56.

^5^EUCAST: Expert rules and expected phenotypes. Accessed Feb 21, 2025. https://www.eucast.org/expert_rules_and_expected_phenotypes.

^6^PubMLST: *Haemophilus influenzae*. Accessed Feb 21, 2025. https://pubmlst.org/organisms/haemophilus-influenzae.

^7^NORM: Surveillance reports. Accessed Feb 21, 2025. https://www.unn.no/fag-og-forskning/norm-norsk-overvakingssystem-for-antibiotikaresistens-hos-mikrober#rapporter.

^8^Statistics Norway: Population. Accessed Feb 21, 2025. https://www.ssb.no/en/statbank/table/07459.

^9^EUCAST: MIC determination. Accessed Feb 21, 2025. https://www.eucast.org/ast_of_bacteria/mic_determination.

^10^EUCAST: MIC and zone diameter distributions and ECOFFs. Accessed Feb 21, 2025. https://www.eucast.org/mic_and_zone_distributions_and_ecoffs.

^11^EUCAST: Quality control. Accessed Feb 21, 2025. https://www.eucast.org/ast_of_bacteria/quality_control.

^12^EUCAST: Definitions of S, I and R. Accessed Feb 21, 2025. https://www.eucast.org/newsiandr.

^13^GitHub: SPAdes. Accessed Feb 21, 2025. https://github.com/ablab/spades.

^14^GitHub: Prokka. Accessed Feb 21, 2025. https://github.com/tseemann/prokka.

^15^GitHub: Hicap. Accessed Feb 21, 2025. https://github.com/scwatts/hicap.

^16^GitHub: MLST. Accessed Feb 21, 2025. https://github.com/tseemann/mlst.

^17^Bac/Pop: PopPUNK databases – *Haemophilus influenzae* (v2). Accessed Feb 21, 2025. https://www.bacpop.org/poppunk/.

^18^GitHub: Panaroo. Accessed Feb 21, 2025. https://github.com/gtonkinhill/panaroo.

^19^GitHub: RaxmlGUI. Accessed Feb 21, 2025. https://github.com/antonellilab/raxmlGUI.

^20^Microreact. Accessed Feb 21, 2025. https://microreact.org.

^21^GitHub: Minimap2. Accessed Feb 21, 2025. https://github.com/lh3/minimap2.

^22^PubMLST: GrapeTree. Accessed Feb 21, 2025. https://pubmlst.org/GrapeTree/MSTree_holder.html.

^23^GitHub: MSA. Accessed Feb 21, 2025. https://github.com/UBod/msa.

^24^GitHub: MAFFT. Accessed Feb 21, 2025. https://github.com/GSLBiotech/mafft.

^25^PubMLST: *ftsI* sequencing protocol. Accessed Feb 21, 2025. https://pubmlst.org/static/organisms/haemophilus-influenzae/ftsI_sequencing_protocol_v3_01-02-2014.pdf.

^26^GitHub: ABRIcate. Accessed Feb 21, 2025. https://github.com/tseemann/abricate.

^27^CGE: ResFinder. Accessed Feb 21, 2025. https://genepi.food.dtu.dk/resfinder.

^28^NCBI: AMRFinderPlus. Accessed Feb 21, 2025. https://www.ncbi.nlm.nih.gov/pathogens/antimicrobial-resistance/AMRFinder/.

^29^NCBI: BLAST. Accessed Feb 21, 2025. https://blast.ncbi.nlm.nih.gov.

^30^Statistics kingdom. Accessed Feb 21, 2025. https://www.statskingdom.com.

^31^PubMLST: Locus information – *ftsI*. Accessed Feb 21, 2025. https://pubmlst.org/bigsdb?db=pubmlst_hinfluenzae_seqdef&page=locusInfo&locus=ftsI.

## References

[B1] Abavisani M. Keikha M. Karbalaei M. (2024). First global report about the prevalence of multi-drug resistant *Haemophilus influenzae*: a systematic review and meta-analysis. BMC Infect. Dis. 24:90. 10.1186/s12879-023-08930-5 38225571 PMC10789054

[B2] Bae I. G. Stone G. G. (2019). Activity of ceftaroline against pathogens associated with community-acquired pneumonia collected as part of the AWARE surveillance program, 2015-2016. Diagn. Microbiol. Infect. Dis. 95:114843. 10.1016/j.diagmicrobio.2019.05.015 31416647

[B3] Baker K. S. Jauneikaite E. Hopkins K. L. Lo S. W. Sánchez-BUSÓ L. Getino M.. (2023). Genomics for public health and international surveillance of antimicrobial resistance. Lancet Microbe 4, e1047–e1055. 10.1016/S2666-5247(23)00283-5 37977162

[B4] Bellini D. Koekemoer L. Newman H. Dowson C. G. (2019). Novel and improved crystal structures of *H. influenzae, E. coli* and *P. aeruginosa* penicillin-binding protein 3 (PBP3) and *N. gonorrhoeae* PBP2: toward a better understanding of β-lactam target-mediated resistance. J. Mol. Biol. 431, 3501–3519. 10.1016/j.jmb.2019.07.010 31301409

[B5] Blázquez J. Couce A. Rodríguez-Beltrán J. Rodríguez-ROJAS A. (2012). Antimicrobials as promoters of genetic variation. Curr. Opin. Microbiol. 15, 561–569. 10.1016/j.mib.2012.07.007 22890188

[B6] Blix H. S. Vestrheim D. F. Hjellvik V. Skaare D. Christensen A. Steinbakk M. (2015). Antibiotic prescriptions and cycles of *Mycoplasma pneumoniae* infections in Norway: can a nationwide prescription register be used for surveillance? Epidemiol. Infect. 143, 1884–1892. 10.1017/S0950268814002908 25388750 PMC4456768

[B7] Caméléna F. Merimèche M. Liberge M. Maubaret C. Donay J. L. Taha M. K.. (2024). Detection of CTX-M-15 ESBL in XDR *Haemophilus parainfluenzae* from a urethral swab. J. Antimicrob. Chemother. 79, 539–545. 10.1093/jac/dkad408 38197448

[B8] Carrera-Salinas A. González-Díaz A. Ehrlich R. L. Berbel D. Tubau F. Pomares X.. (2023). Genetic adaptation and acquisition of macrolide resistance in *Haemophilus* spp. during persistent respiratory tract colonization in chronic obstructive pulmonary disease (COPD) patients receiving long-term azithromycin treatment. Microbiol. Spectr. 11:e0386022. 10.1128/spectrum.03860-22 36475849 PMC9927455

[B9] Cherkaoui A. Francois P. Gaia N. Renzi G. Fischer A. Schrenzel J. (2025). Extensively drug-resistant *Haemophilus influenzae* isolated in Geneva, Switzerland. Eur. J. Clin. Microbiol. Infect. Dis. 44, 1273–1277. 10.1007/s10096-025-05093-w 40048099 PMC12062098

[B10] Connor T. R. Corander J. Hanage W. P. (2012). Population subdivision and the detection of recombination in non-typable *Haemophilus influenzae*. Microbiology 158, 2958–2964. 10.1099/mic.0.063073-0 23038806 PMC4083659

[B11] De Chiara M. Hood D. Muzzi A. Pickard D. J. Perkins T. Pizza M.. (2014). Genome sequencing of disease and carriage isolates of nontypeable *Haemophilus influenzae* identifies discrete population structure. Proc. Natl. Acad. Sci. U. S. A. 111, 5439–5444. 10.1073/pnas.1403353111 24706866 PMC3986186

[B12] Deghmane A. E. Hong E. Chehboub S. Terrade A. Falguières M. Sort M.. (2019). High diversity of invasive *Haemophilus influenzae* isolates in France and the emergence of resistance to third generation cephalosporins by alteration of *ftsI* gene. J. Infect. 79, 7–14. 10.1016/j.jinf.2019.05.007 31100360

[B13] Diricks M. Petersen S. Bartels L. Lâm T. T. Claus H. Bajanca-Lavado M. P.. (2024). Revisiting mutational resistance to ampicillin and cefotaxime in *Haemophilus influenzae*. Genome Med. 16:140. 10.1186/s13073-024-01406-4 39633433 PMC11616347

[B14] El Nouwar R. Prevost B. Wautier M. Yin N. Hites M. Martiny D. (2025). Epidemiology of invasive *Haemophilus influenzae* infections in Belgium: 2018-2022. Eur. J. Clin. Microbiol. Infect. Dis. 44, 855–865. 10.1007/s10096-025-05040-9 39875613

[B15] Fukasawa C. Hoshino T. Kutsuna S. Sawada K. Sato H. Ishiwada N. (2013). [Concentration of tazobactam/piperacillin in the cerebrospinal fluid of patients with *Haemophilus influenzae* type B meningitis]. Kansenshogaku Zasshi 87, 590–595. 10.11150/kansenshogakuzasshi.87.590 24195168

[B16] García-Cobos S. Campos J. Lázaro E. Román F. Cercenado E. García-Rey C.. (2007). Ampicillin-resistant non-beta-lactamase-producing *Haemophilus influenzae* in Spain: recent emergence of clonal isolates with increased resistance to cefotaxime and cefixime. Antimicrob. Agents Chemother. 51, 2564–2573. 10.1128/AAC.00354-07 17470649 PMC1913223

[B17] García-Rodríguez J. A. Fresnadillo Martínez M. J. (2002). Dynamics of nasopharyngeal colonization by potential respiratory pathogens. J. Antimicrob. Chemother. 50(Suppl.S2), 59–73. 10.1093/jac/dkf506 12556435

[B18] Hegstad K. Mylvaganam H. Janice J. Josefsen E. Sivertsen A. Skaare D. (2020). Role of horizontal gene transfer in the development of multidrug resistance in *Haemophilus influenzae*. mSphere 5:e00969-19. 10.1128/mSphere.00969-19 31996416 PMC6992377

[B19] Jakubu V. Vrbova I. Bitar I. Cechova M. Malisova L. Zemlickova H. (2024). Evolution of mutations in the *ftsI* gene leading to amino acid substitutions in PBP3 in *Haemophilus influenzae* strains under the selective pressure of ampicillin and cefuroxime. Int. J. Med. Microbiol. 316:151626. 10.1016/j.ijmm.2024.151626 38954914

[B20] Johannessen H. Anthonisen I. L. Zecic N. Hegstad K. Ranheim T. E. Skaare D. (2022). Characterization and fitness cost of Tn*7100*, a novel integrative and conjugative element conferring multidrug resistance in *Haemophilus influenzae*. Front. Microbiol. 13:945411. 10.3389/fmicb.2022.945411 35935209 PMC9355037

[B21] Jolley K. A. Bray J. E. Maiden M. C. J. (2018). Open-access bacterial population genomics: BIGSdb software, the PubMLST.org website and their applications. Wellcome Open Res. 3:124. 10.12688/wellcomeopenres.14826.1 30345391 PMC6192448

[B22] Juhas M. Power P. M. Harding R. M. Ferguson D. J. Dimopoulou I. D. Elamin A. R.. (2007). Sequence and functional analyses of *Haemophilus* spp. genomic islands. Genome Biol. 8:R237. 10.1186/gb-2007-8-11-r237 17996041 PMC2258188

[B23] Krisna M. A. Jolley K. A. Monteith W. Boubour A. Hamers R. L. Brueggemann A. B.. (2024). Development and implementation of a core genome multilocus sequence typing scheme for *Haemophilus influenzae*. Microb. Genom. 10:001281. 10.1099/mgen.0.001281 39120932 PMC11315579

[B24] Lees J. A. Harris S. R. Tonkin-Hill G. Gladstone R. A. Lo S. W. Weiser J. N.. (2019). Fast and flexible bacterial genomic epidemiology with PopPUNK. Genome Res. 29, 304–316. 10.1101/gr.241455.118 30679308 PMC6360808

[B25] Li X. Mariano N. Rahal J. J. Urban C. M. Drlica K. (2004). Quinolone-resistant *Haemophilus influenzae*: determination of mutant selection window for ciprofloxacin, garenoxacin, levofloxacin, and moxifloxacin. Antimicrob. Agents Chemother. 48, 4460–4462. 10.1128/AAC.48.11.4460-4462.2004 15504883 PMC525419

[B26] Magiorakos A. P. Srinivasan A. Carey R. B. Carmeli Y. Falagas M. E. Giske C. G.. (2012). Multidrug-resistant, extensively drug-resistant and pandrug-resistant bacteria: an international expert proposal for interim standard definitions for acquired resistance. Clin. Microbiol. Infect. 18, 268–281. 10.1111/j.1469-0691.2011.03570.x 21793988

[B27] Meats E. Feil E. J. Stringer S. Cody A. J. Goldstein R. Kroll J. S.. (2003). Characterization of encapsulated and noncapsulated Haemophilus influenzae and determination of phylogenetic relationships by multilocus sequence typing. J. Clin. Microbiol. 41, 1623–1636. 10.1128/JCM.41.4.1623-1636.2003 12682154 PMC153921

[B28] Mell J. C. Hall I. M. Redfield R. J. (2012). Defining the DNA uptake specificity of naturally competent Haemophilus influenzae cells. Nucleic Acids Res. 40, 8536–8549. 10.1093/nar/gks640 22753031 PMC3458573

[B29] Merlino J. Rizzo S. English S. Baskar S. R. Siarakas S. Mckew G.. (2023). *Haemophilus influenzae* blood-stream infection and third-generation cephalosporin susceptibility testing: a comparative case study using EUCAST and CLSI guidelines. Access Microbiol. 5:000578.v4. 10.1099/acmi.0.000578.v4 37970074 PMC10634490

[B30] Michel C. Argudín M. A. Wautier M. Echahidi F. Prevost B. Vandenberg O.. (2024). Multiple interspecies recombination events documented by whole-genome sequencing in multidrug-resistant *Haemophilus influenzae* clinical isolates. Access Microbiol. 6:000649.v3. 10.1099/acmi.0.000649.v3 38482359 PMC10928409

[B31] Mizoguchi A. Hitomi S. (2019). Cefotaxime-non-susceptibility of *Haemophilus influenzae* induced by additional amino acid substitutions of G555E and Y557H in altered penicillin-binding protein 3. J. Infect. Chemother. 25, 509–513. 10.1016/j.jiac.2019.02.010 30879978

[B32] Nørskov-Lauritsen N. (2014). Classification, identification, and clinical significance of *Haemophilus* and *Aggregatibacter* species with host specificity for humans. Clin. Microbiol. Rev. 27, 214–240. 10.1128/CMR.00103-13 24696434 PMC3993099

[B33] Nürnberg S. Claus H. Krone M. Vogel U. Lâm T. T. (2021). Cefotaxime resistance in invasive *Haemophilus influenzae* isolates in Germany 2016-19: prevalence, epidemiology and relevance of PBP3 substitutions. J. Antimicrob. Chemother. 76, 920–929. 10.1093/jac/dkaa557 33501993

[B34] Osaki Y. Sanbongi Y. Ishikawa M. Kataoka H. Suzuki T. Maeda K.. (2005). Genetic approach to study the relationship between penicillin-binding protein 3 mutations and *Haemophilus influenzae* beta-lactam resistance by using site-directed mutagenesis and gene recombinants. Antimicrob. Agents Chemother. 49, 2834–2839. 10.1128/AAC.49.7.2834-2839.2005 15980357 PMC1168665

[B35] Regelink A. G. Dahan D. Möller L. V. Coulton J. W. Eijk P. Van Ulsen P.. (1999). Variation in the composition and pore function of major outer membrane pore protein P2 of *Haemophilus influenzae* from cystic fibrosis patients. Antimicrob. Agents Chemother. 43, 226–232. 10.1128/AAC.43.2.226 9925510 PMC89055

[B36] Saiz-Escobedo L. Cadenas-Jiménez I. Olmos R. Carrera-Salinas A. Berbel D. Càmara J.. (2023). Detection of *bla*(CTX-M-15) in an integrative and conjugative element in four extensively drug-resistant *Haemophilus parainfluenzae* strains causing urethritis. Int. J. Antimicrob. Agents 62:106991. 10.1016/j.ijantimicag.2023.106991 37774891

[B37] Skaare D. Anthonisen I. L. Caugant D. A. Jenkins A. Steinbakk M. Strand L.. (2014a). Multilocus sequence typing and *ftsI* sequencing: a powerful tool for surveillance of penicillin-binding protein 3-mediated beta-lactam resistance in nontypeable *Haemophilus influenzae*. BMC Microbiol. 14:131. 10.1186/1471-2180-14-131 24884375 PMC4039647

[B38] Skaare D. Anthonisen I. L. Kahlmeter G. Matuschek E. Natas O. B. Steinbakk M.. (2014b). Emergence of clonally related multidrug resistant *Haemophilus influenzae* with penicillin-binding protein 3-mediated resistance to extended-spectrum cephalosporins, Norway, 2006 to 2013. Euro Surveill. 19:20986. 10.2807/1560-7917.ES2014.19.49.20986 25523969

[B39] Skaare D. Lia A. Hannisdal A. Tveten Y. Matuschek E. Kahlmeter G.. (2015). *Haemophilus influenzae* with non-beta-lactamase-mediated beta-lactam resistance: easy to find but hard to categorize. J. Clin. Microbiol. 53, 3589–3595. 10.1128/JCM.01630-15 26354813 PMC4609732

[B40] Slack M. P. E. Cripps A. W. Grimwood K. Mackenzie G. A. Ulanova M. (2021). Invasive *Haemophilus influenzae* infections after 3 decades of Hib protein conjugate vaccine use. Clin. Microbiol. Rev. 34:e0002821. 10.1128/CMR.00028-21 34076491 PMC8262803

[B41] Smith J. M. Smith N. H. O'Rourke M. Spratt B. G. (1993). How clonal are bacteria? Proc. Natl. Acad. Sci. U. S. A. 90, 4384–4388. 10.1073/pnas.90.10.4384 8506277 PMC46515

[B42] Taha A. Adeline F. Taha M. K. Deghmane A. E. (2022). *Haemophilus influenzae* drug resistance in France from 2017 to 2021: consideration for treatment of otitis media. J. Glob. Antimicrob. Resist. 31, 222–227. 10.1016/j.jgar.2022.09.008 36195280

[B43] Takahata S. Ida T. Senju N. Sanbongi Y. Miyata A. Maebashi K.. (2007). Horizontal gene transfer of *ftsI*, encoding penicillin-binding protein 3, in *Haemophilus influenzae*. Antimicrob. Agents Chemother. 51, 1589–1595. 10.1128/AAC.01545-06 17325223 PMC1855551

[B44] Thegerström J. Matuschek E. Su Y. C. Riesbeck K. Resman F. (2018). A novel PBP3 substitution in *Haemophilus influenzae* confers reduced aminopenicillin susceptibility. BMC Microbiol. 18:48. 10.1186/s12866-018-1196-6 29855260 PMC5984330

[B45] Tonkin-Hill G. Macalasdair N. Ruis C. Weimann A. Horesh G. Lees J. A.. (2020). Producing polished prokaryotic pangenomes with the Panaroo pipeline. Genome Biol. 21:180. 10.1186/s13059-020-02090-4 32698896 PMC7376924

[B46] Tønnessen R. García I. Debech N. Lindstrøm J. C. Wester A. L. Skaare D. (2022). Molecular epidemiology and antibiotic resistance profiles of invasive *Haemophilus influenzae* from Norway 2017–2021. Front. Microbiol. 13:973257. 10.3389/fmicb.2022.973257 36106084 PMC9467436

[B47] Tristram S. Jacobs M. R. Appelbaum P. C. (2007). Antimicrobial resistance in *Haemophilus influenzae*. Clin. Microbiol. Rev. 20, 368–389. 10.1128/CMR.00040-06 17428889 PMC1865592

[B48] Ubukata K. (2003). Problems associated with high prevalence of multidrug-resistant bacteria in patients with community-acquired infections. J. Infect. Chemother. 9, 285–291. 10.1007/s10156-003-0278-Y 14691647

[B49] Ubukata K. Shibasaki Y. Yamamoto K. Chiba N. Hasegawa K. Takeuchi Y.. (2001). Association of amino acid substitutions in penicillin-binding protein 3 with beta-lactam resistance in beta-lactamase-negative ampicillin-resistant *Haemophilus influenzae*. Antimicrob. Agents Chemother. 45, 1693–1699. 10.1128/AAC.45.6.1693-1699.2001 11353613 PMC90533

[B50] Van Eldere J. Slack M. P. Ladhani S. Cripps A. W. (2014). Non-typeable *Haemophilus influenzae*, an under-recognised pathogen. Lancet Infect. Dis. 14, 1281–1292. 10.1016/S1473-3099(14)70734-0 25012226

[B51] Vinatzer B. A. Tian L. Heath L. S. (2017). A proposal for a portal to make earth's microbial diversity easily accessible and searchable. Antonie Van Leeuwenhoek 110, 1271–1279. 10.1007/s10482-017-0849-z 28281028

[B52] Wienholtz N. H. Barut A. Nørskov-Lauritsen N. (2017). Substitutions in PBP3 confer resistance to both ampicillin and extended-spectrum cephalosporins in *Haemophilus parainfluenzae* as revealed by site-directed mutagenesis and gene recombinants. J. Antimicrob. Chemother. 72, 2544–2547. 10.1093/jac/dkx157 28582518

[B53] Witherden E. A. Bajanca-Lavado M. P. Tristram S. G. Nunes A. (2014). Role of inter-species recombination of the *ftsI* gene in the dissemination of altered penicillin-binding-protein-3-mediated resistance in *Haemophilus influenzae* and *Haemophilus haemolyticus*. J. Antimicrob. Chemother. 69, 1501–1509. 10.1093/jac/dku022 24562614

[B54] Woodford N. Turton J. F. Livermore D. M. (2011). Multiresistant Gram-negative bacteria: the role of high-risk clones in the dissemination of antibiotic resistance. FEMS Microbiol. Rev. 35, 736–755. 10.1111/j.1574-6976.2011.00268.x 21303394

