# Supplementary material for: Genomic profiling of cefotaxime-resistant Haemophilus influenzae from Norway and Sweden reveals extensive expansion of virulent multidrug-resistant international clones
Source: Front Microbiol. 2025 Jul 29;16:1601390. doi: 10.3389/fmicb.2025.1601390 (PMC12341455; doi:10.3389/fmicb.2025.1601390)

**Supplementary Figure S1.** Selection and inclusion. *H. influenzae* with cefotaxime gradient MIC (CTX gMIC) > 0.125 mg/L were collected from three sources: 1) routine diagnostics at Vestfold Hospital Trust (VHT), 2) isolates with ‘exceptional phenotypes’ submitted to VHT, and 3) the Norwegian Surveillance System for Antimicrobial Drug Resistance (NORM). Isolates not growing in the broth microdilution MIC assay and isolates falsely cefotaxime-resistant by gradient MIC underwent sequencing to allow classification of penicillin-binding protein 3-mediated resistance (rPBP3) but were otherwise excluded from further analyses. Overall, 191 isolates from different patients were confirmed cefotaxime-resistant *H. influenzae* (CRHI) and included in the study.


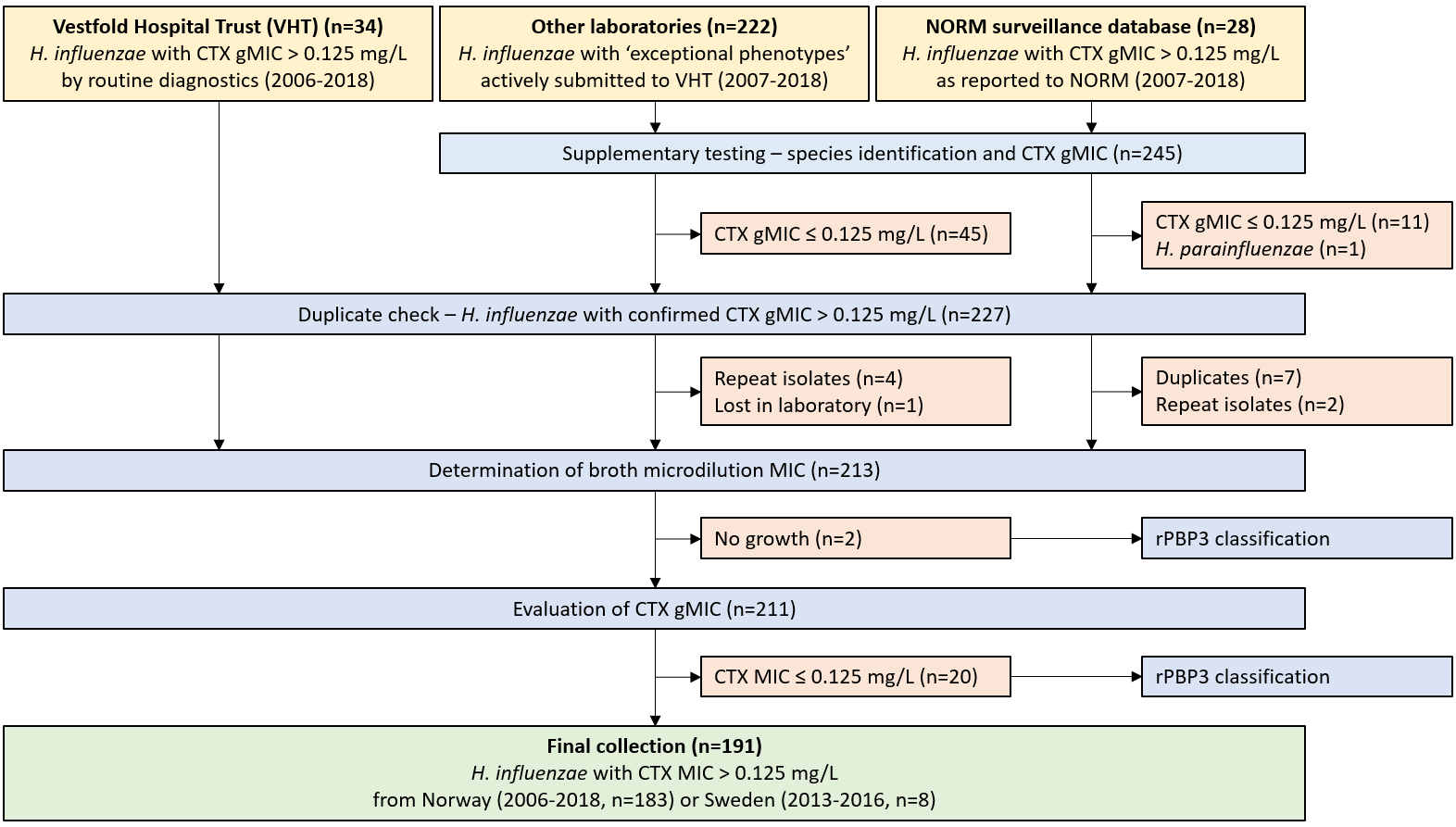


**Supplementary Figure S2.** Geographical distribution. A) Norwegian patients (n=183) were assigned to six geographical regions based on county of residence: Capital (Oslo County), East (Akershus, Innlandet, and Østfold counties), South (Agder, Buskerud, Telemark, and Vestfold counties), West (Rogaland and Vestland counties), Central (Møre og Romsdal and Trøndelag counties), and North (Finnmark, Nordland, and Troms counties). Sweden (n=8) was counted as a single region. B) Included isolates (n=191) by region and year. The low number in 2018 reflects downscaling of confirmatory testing at Vestfold Hospital Trust due to altered EUCAST recommendations for handling of *H. influenzae* with exceptional phenotypes. C) Isolates by region and care level (primary care or hospital). Proportions of the Norwegian population (grey columns) are shown as comparators.


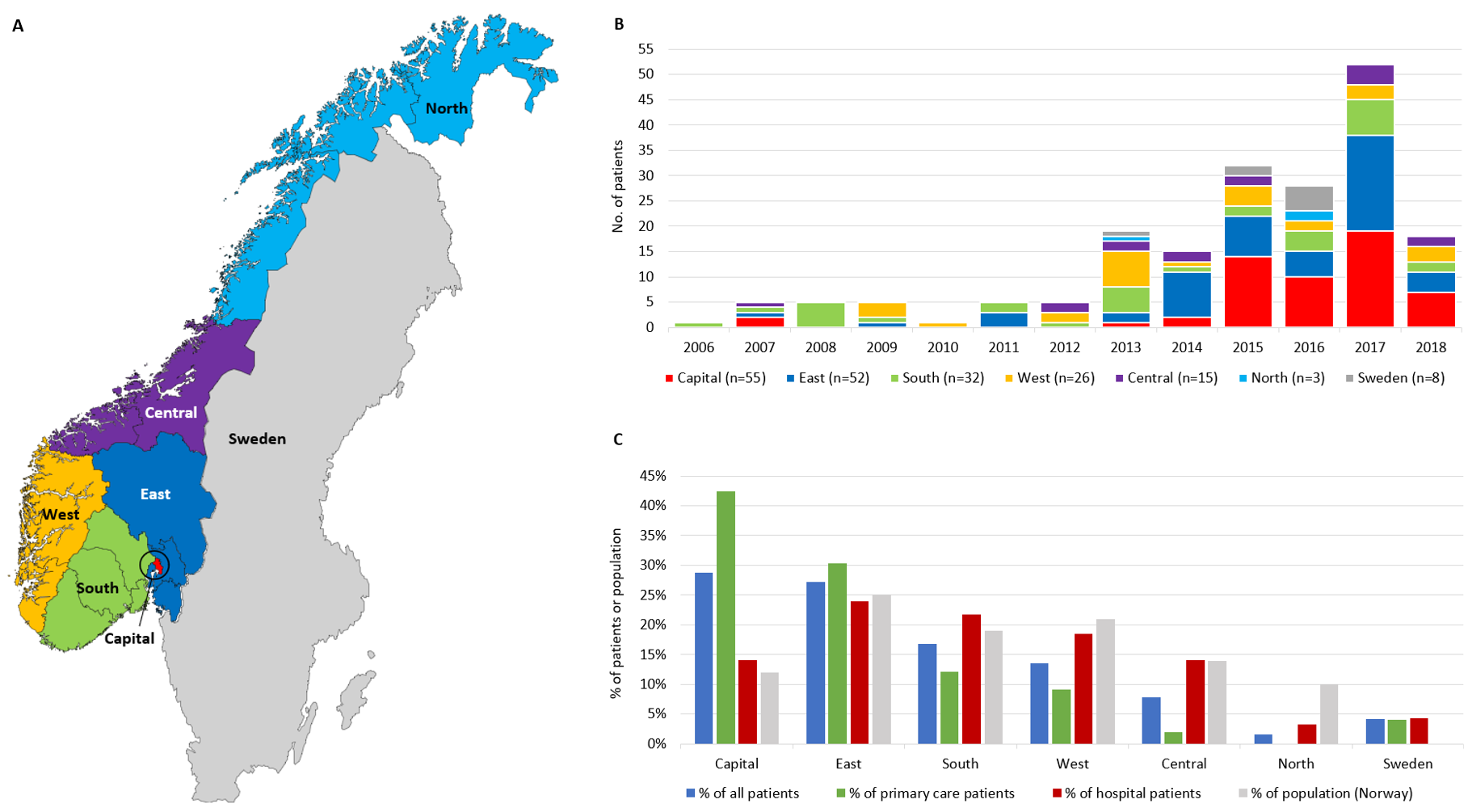


**Supplementary Table S1.** Antimicrobial agents and categories, epidemiological cuff-off values (ECOFF), and clinical breakpoints

| **Antimicrobial categories^a^** | **Agents** | **Epidemiologically significant^b^** | **BMD MIC calling range (mg/L)** | **ECOFF**  **(mg/L)^c^** | **Breakpoints**  **(mg/L)^d^** |
| --- | --- | --- | --- | --- | --- |
| Aminopenicillins | Ampicillin | Yes | 0.5 – 16 | 1 | 1/1 |
| Aminopenicillins w/ BLI | Ampicillin-sulbactam | Yes | 0.5 – 16^e^ | 1 | 1/1 |
|  | Amoxicillin-clavulanic acid (iv) | Yes | 1 – 32^e^ | 2 | 2/2 |
| Antipseudomonal penicillin w/ BLI | Piperacillin-tazobactam | Yes | 0.06 – 4^e^ | 0.125 | 0.25/0.25 |
| Extended-spectrum cephalosporins | Cefotaxime | Yes | 0.06 – 8 | 0.06 | 0.125/0.125 |
|  | Ceftriaxone | Yes | 0.06 – 4 | None | 0.125/0.125 |
|  | Cefepime | Yes | 0.25 – 4 | 0.25 | 0.25/0.25 |
| Carbapenems | Meropenem (general breakpoints) | Yes | 0.25 – 4 | 0.125 | 2/2 |
|  | Meropenem (meningitis breakpoints) | Yes | 0.25 – 4 | 0.125 | 0.25/0.25 |
|  | Imipenem | Yes | 2 – 4 | 4 | 2/2 |
| Quinolones | Ciprofloxacin (general breakpoints) | Yes | 0.06 – 2 | 0.03 | 0.06/0.06 |
|  | Levofloxacin | Yes | 0.06 – 2 | 0.06 | 0.06/0.06 |
|  | Moxifloxacin | Yes | 0.125 – 1 | 0.125 | 0.125/0.125 |
| Phenicols | Chloramphenicol | Yes | 1 – 8 | 2 | 2/2 |
| Folate pathway inhibitors | Trimethoprim-sulfamethoxazole^f^ | Yes | 0.5 – 4 | 0.5 | 0.5/1 |
| Tetracyclines | Doxycycline | Yes | 0.5 – 2 | 1 | 1/1 |
|  | Tetracycline | Yes | 1 – 8 | 1 | 2/2 |
|  | Minocycline | Yes | 1 – 2 | 1 | 1/1 |
| Rifamycines | Rifampicin (for prophylaxis only) | No | 1 – 2 | 1 | 1/1 |
| Macrolides | Azithromycin | No | 4 – 32 | 4 | None |
| Glycylcyclines | Tigecycline | No | 1 | 1 | None |
| Aminoglycosides | Gentamicin | No | 4 | None^g^ | None |

^a^According to principles proposed by Magiorakos et al., 2012. BLI, beta-lactamase inhibitor

^b^Defined as agents with breakpoints for therapeutic use as defined by the European Committee on Antimicrobial Susceptibility Testing (EUCAST)

^c^ECOFFs as defined by EUCAST. The concentrations indicate the highest MIC for isolates without acquired resistance

^d^EUCAST clinical breakpoints (v. 14.0, 2024). The breakpoints are presented as susceptible (S) ≤ / resistant (R) >

^e^Broth microdilution (BMD) performed with fixed concentrations of clavulanic acid (2 mg/L), sulbactam (4 mg/L), or tazobactam (4 mg/L)

^f^Trimethoprim and sulfamethoxazole in the ratio 1:19. Calling range, ECOFF, and breakpoints are expressed as the trimethoprim concentration

^g^A previous MIC distribution suggested an ECOFF of 4 mg/L (removed from the EUCAST website in 2020)

**Supplementary Figure S3.** Overview of the bioinformatic pipeline. Grey, basic procedures. Blue, molecular typing and phylogeny. Red, detection and characterization of antibiotic resistance. White, output/input files. Dashed lines, subset of isolates.


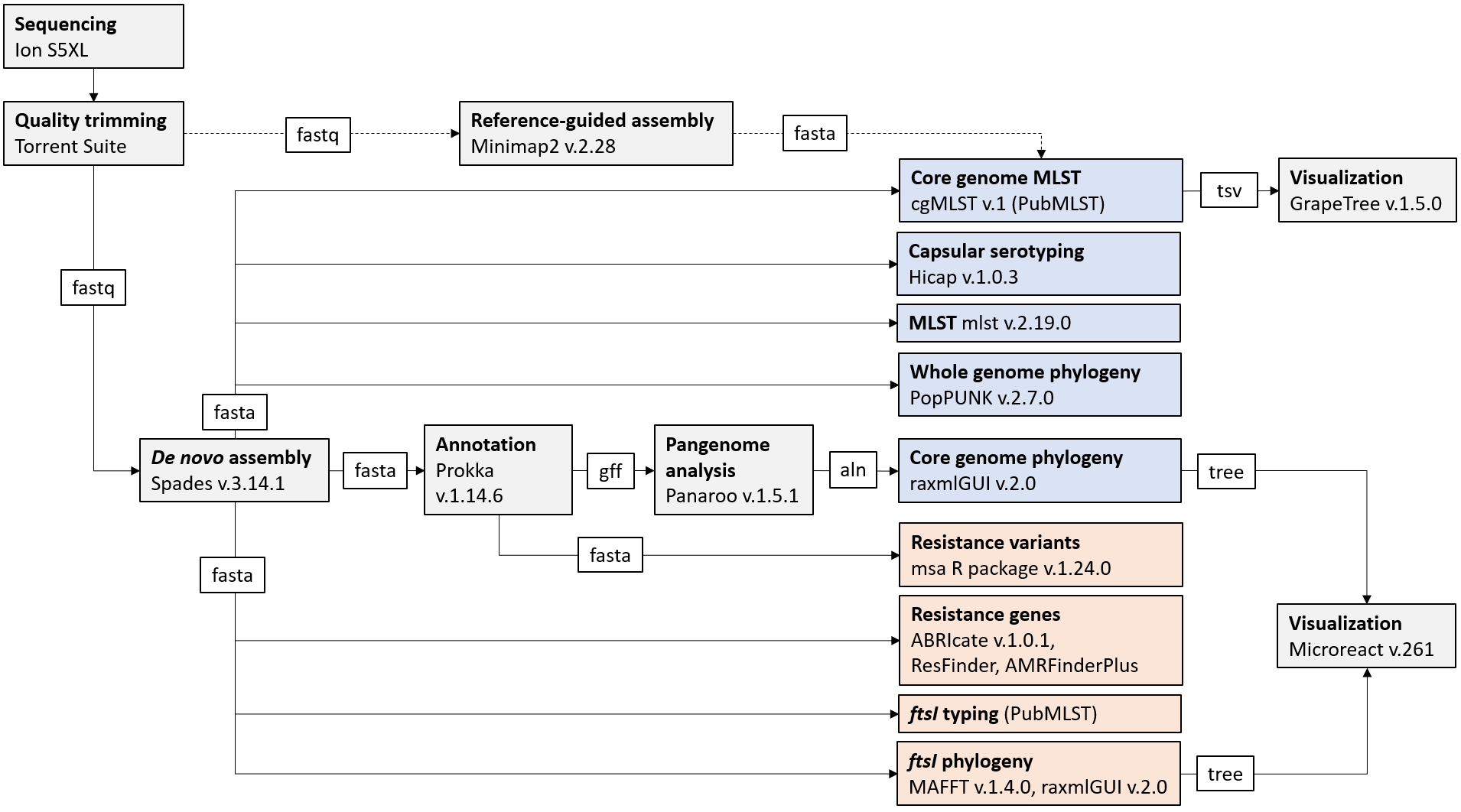


**Supplementary Figure S4.** Patient and sample characteristics (n=191). A) care level, B) age distribution, C) care level with age and sex distribution, D) sample types (overall), E) sample types by care level. RT, respiratory tract.


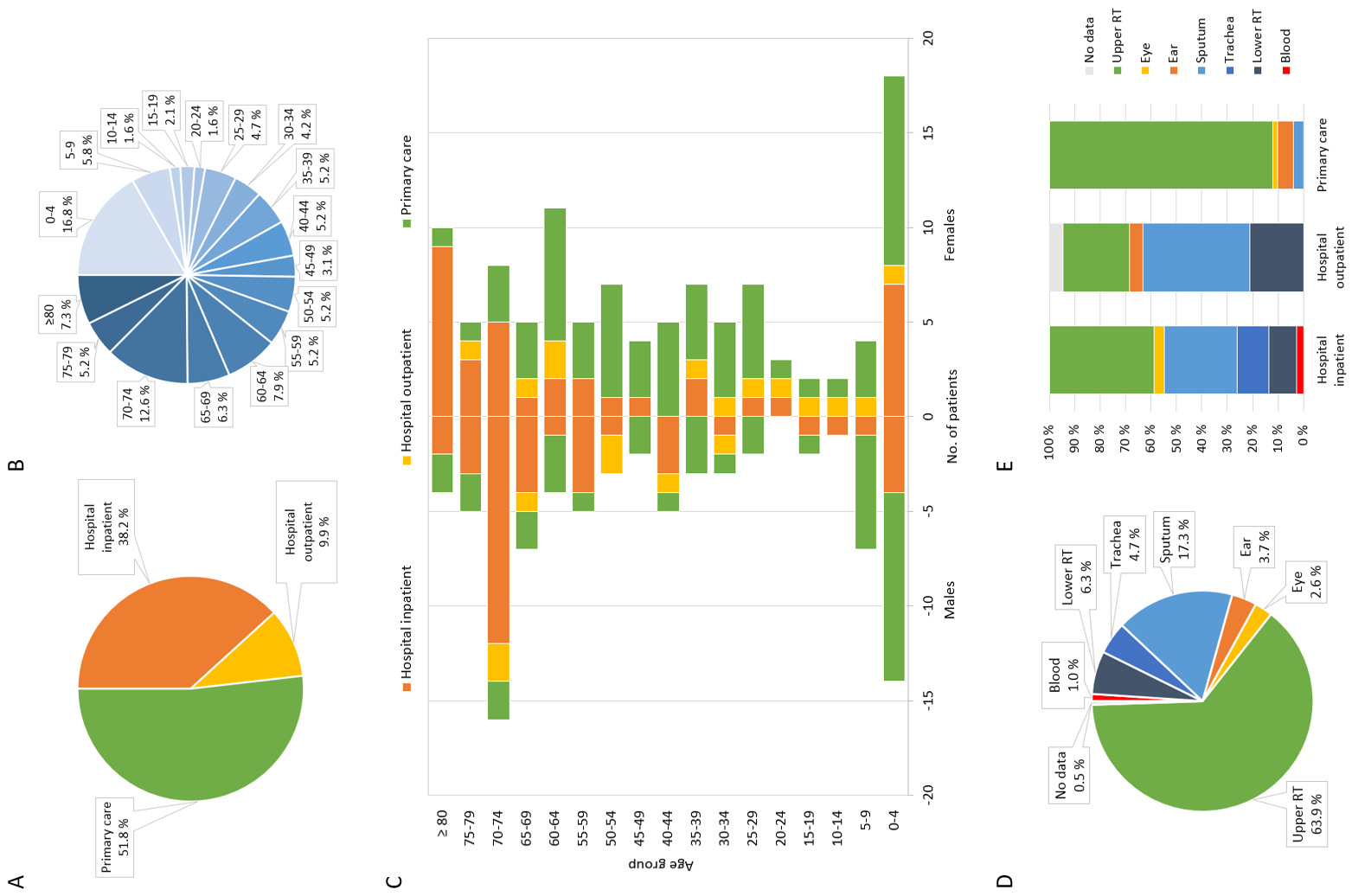


**Supplementary Figure S5.** Isolates reported as cefotaxime-resistant *H. influenzae* (CRHI) to the Norwegian Surveillance System for Antimicrobial Drug Resistance (NORM) (n=28). A) Original cefotaxime gradient MICs (top), gradient MICs obtained in the present study (middle), and broth microdilution (BMD) MICs (bottom). Red and green shades indicate MIC above and below the clinical breakpoint, respectively. Gradient MICs below the horizontal dashed lines (0.25 mg/L) were associated with high likelihood of false resistance (Figure 1). Yellow shade, not *H. influenzae* (Hi). Grey shade, not performed. Proportions of isolates categorized as cefotaxime-resistant were calculated using denominators from the surveillance reports (N). Dash, no isolates. ND, no data. B-C) CRHI prevalence (blue, non-invasive; red, invasive). Filled squares, confirmed with BMD. Plus signs and circles indicate high and low estimates based on surveillance data (gradient MIC > 0.125 mg/L and 0.25 mg/L, respectively).


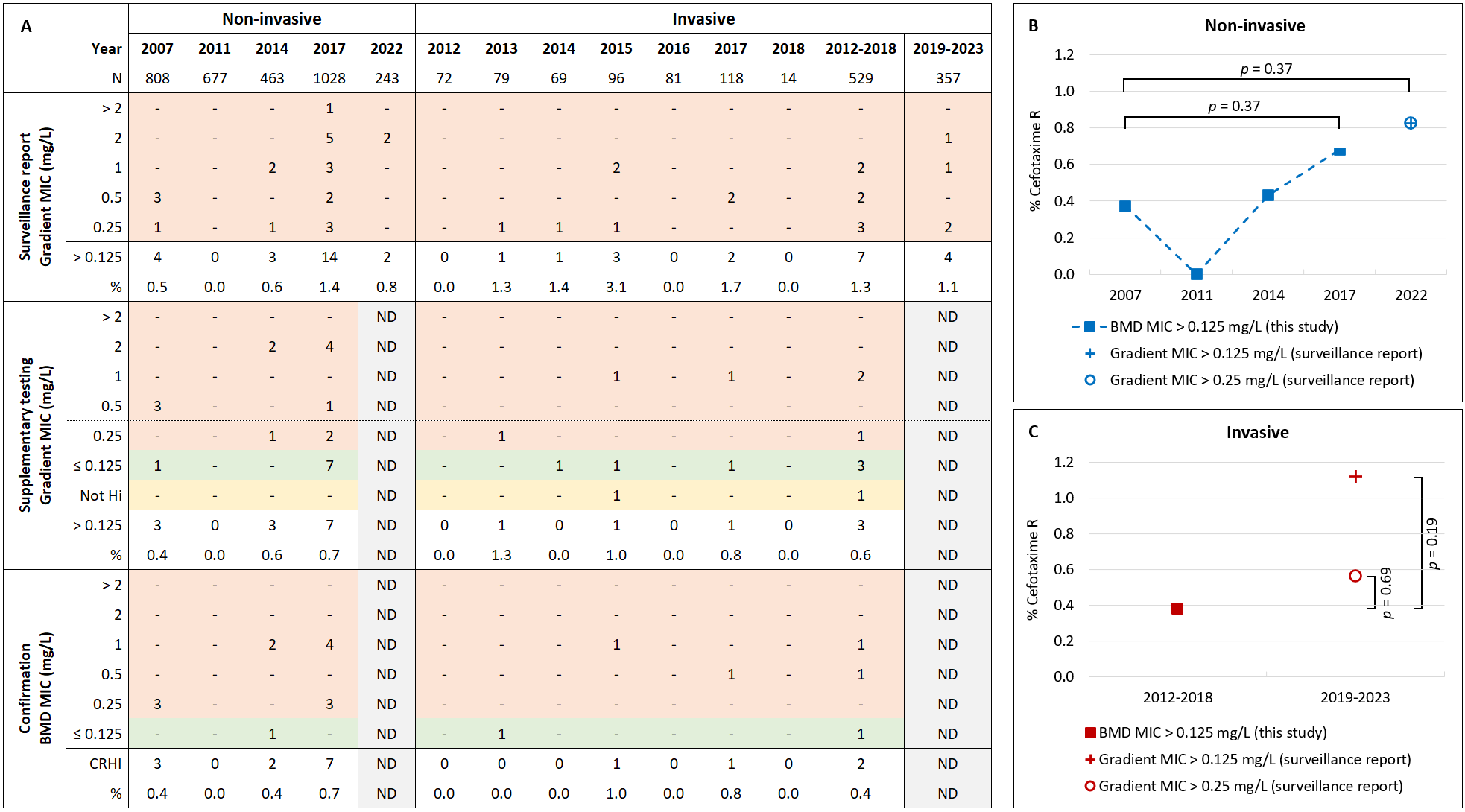


**Supplementary Figure S6.** Phenotypic resistance profiles for the included isolates (n=191). A) selected beta-lactams, B) beta-lactam categories, C) non-beta-lactam categories, and D) classification of multidrug resistance. Colored markers indicate resistance defined as MIC > the resistant (R) breakpoint (red) or MIC > the epidemiological cut-off value (purple). Black dots, susceptible (S) or ‘susceptible, increased exposure’ (I). Categories were labelled ‘R’ if MIC was > the R breakpoint or ECOFF for ≥ 1 agent. Classification of multidrug resistance was based on five beta-lactam and four non-beta-lactam categories comprising agents with breakpoints for therapeutic use (Supplementary Table S1). BLI, beta-lactamase inhibitor. MDR, multidrug-resistant. XDR, extensively drug-resistant.


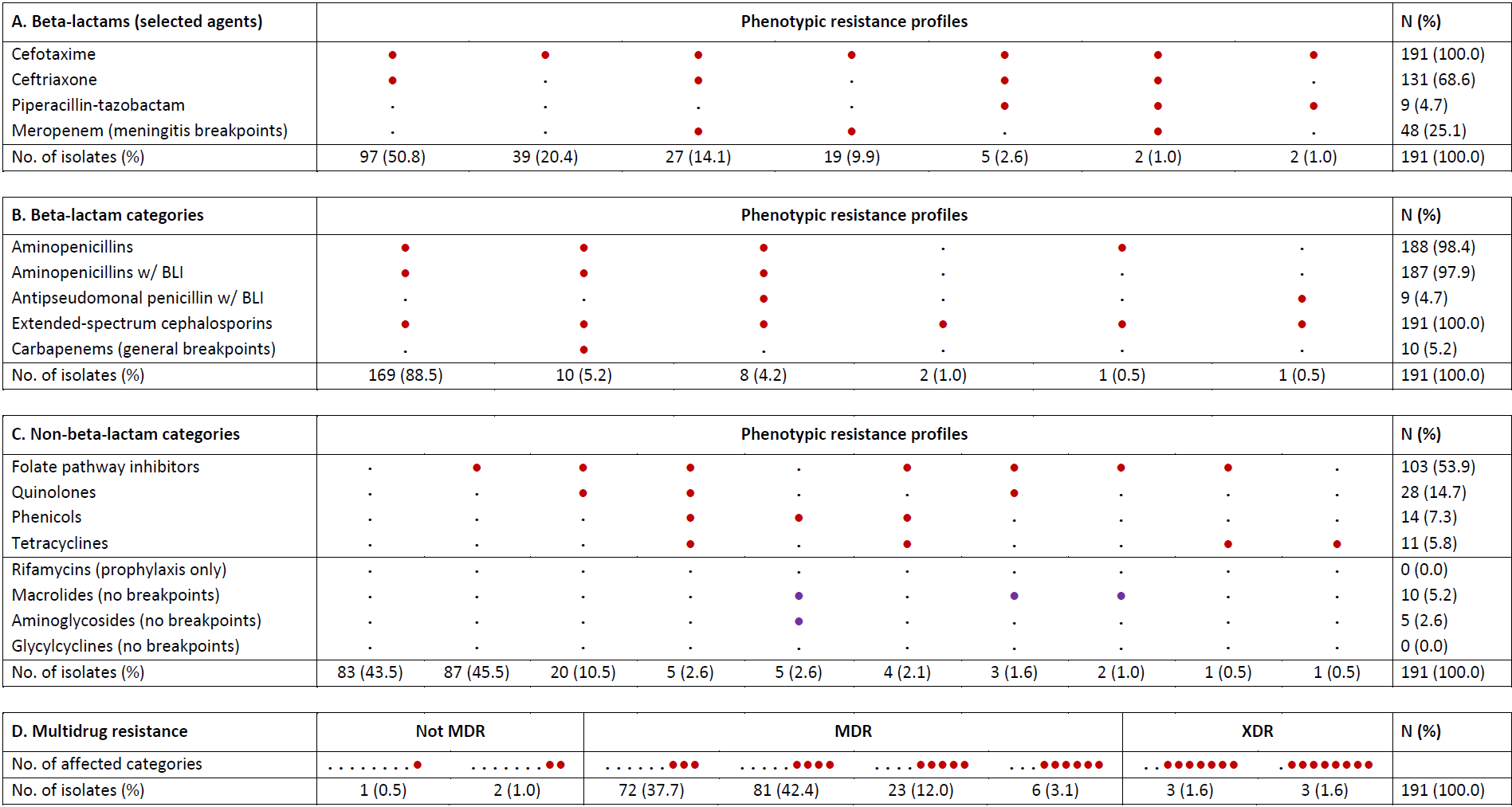


**Supplementary Figure S7.** Minimum spanning tree (MST) based on core genome multilocus sequence typing (cgMLST) of the included isolates (n=191) and *H. influenzae* Rd KW20 ATCC 51907. Node labels and colors indicate clusters (01-27) (same colors as in Figure 4). Transparent nodes, singletons (Sg). Black node, Rd KW20. The clusters represent isolates with identical Life Identification Number (LIN) codes corresponding to a threshold of ≤ 10 allelic differences (LIN9). Four isolates with no LIN code were assigned to clusters 06, 11, and 18 based on core genome phylogeny. Total numbers of isolates in each cluster are shown in the legend [brackets]. Numbers along lines connecting nodes indicate allelic differences (log scale). Larger nodes represent two or more isolates separated by ≤ 10 allelic differences (MST analysis). PopPUNK strains with ≥ 3 representatives (n=16) are encircled (bold numbers outside nodes).


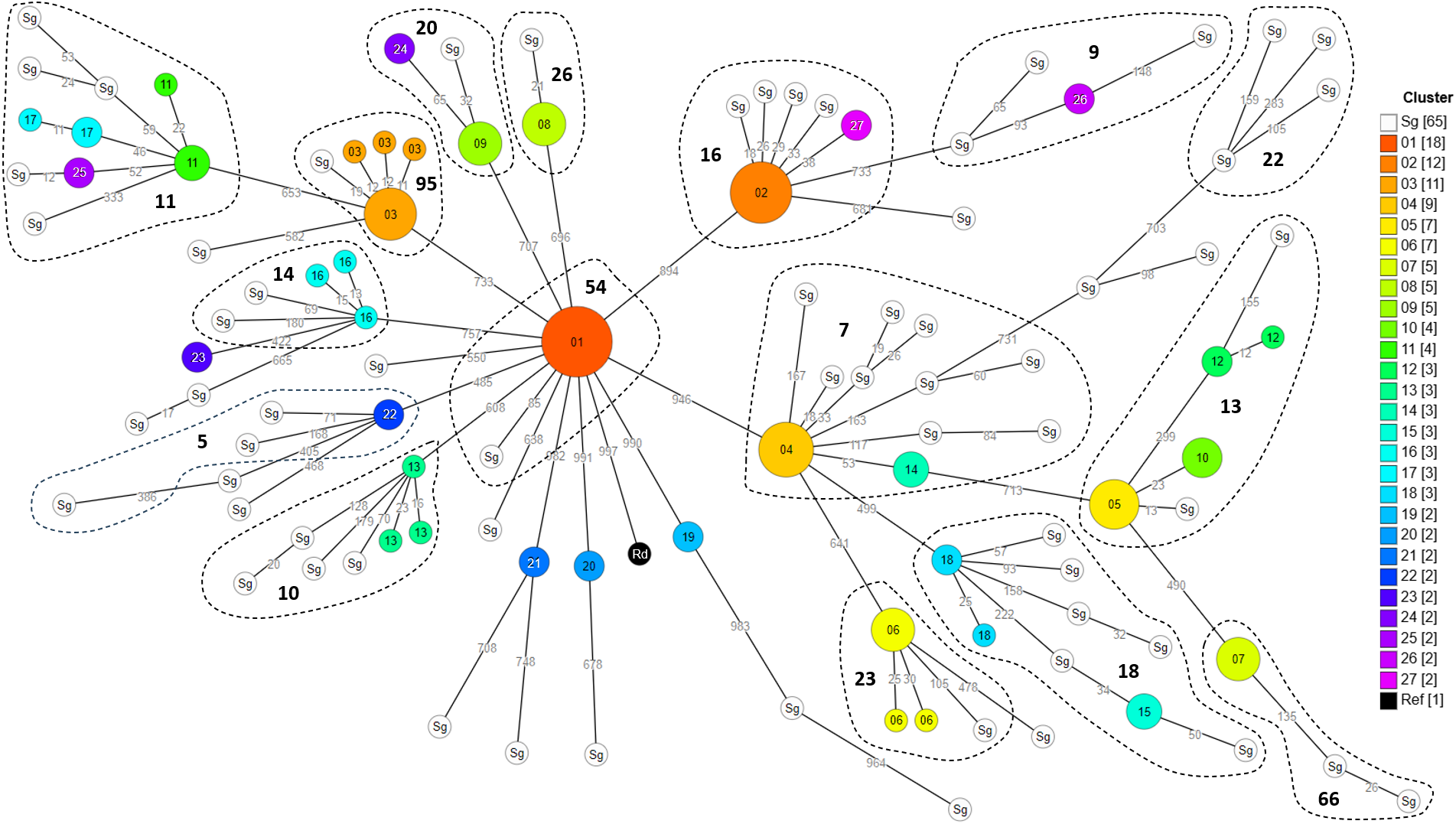


**Supplementary Figure S8.** Minimum spanning tree (MST) based on core genome multilocus sequence typing (cgMLST) of the included isolates (n=191) and *H. influenzae* Rd KW20 ATCC 51907 (same tree as in Supplementary Figure S7). Node colors indicate combinations of PBP3 types and *ftsI* alleles (PBP3-*ftsI* types) present in ≥ 2 isolates (n=15, range 3-35) (same colors as in Figure 6B). Total numbers of isolates with each PBP3-*ftsI* type are shown in the legend [brackets]. Types present in single isolates have transparent nodes (n=34). Node labels indicate clusters (01-27). Sg, singletons. Ref, Rd KW20. Numbers along lines connecting nodes indicate allelic differences (log scale). Larger nodes represent ≥ 2 isolates separated by ≤ 10 allelic differences (MST analysis). PopPUNK strains with ≥ 3 representatives (n=16) are encircled (bold labels).


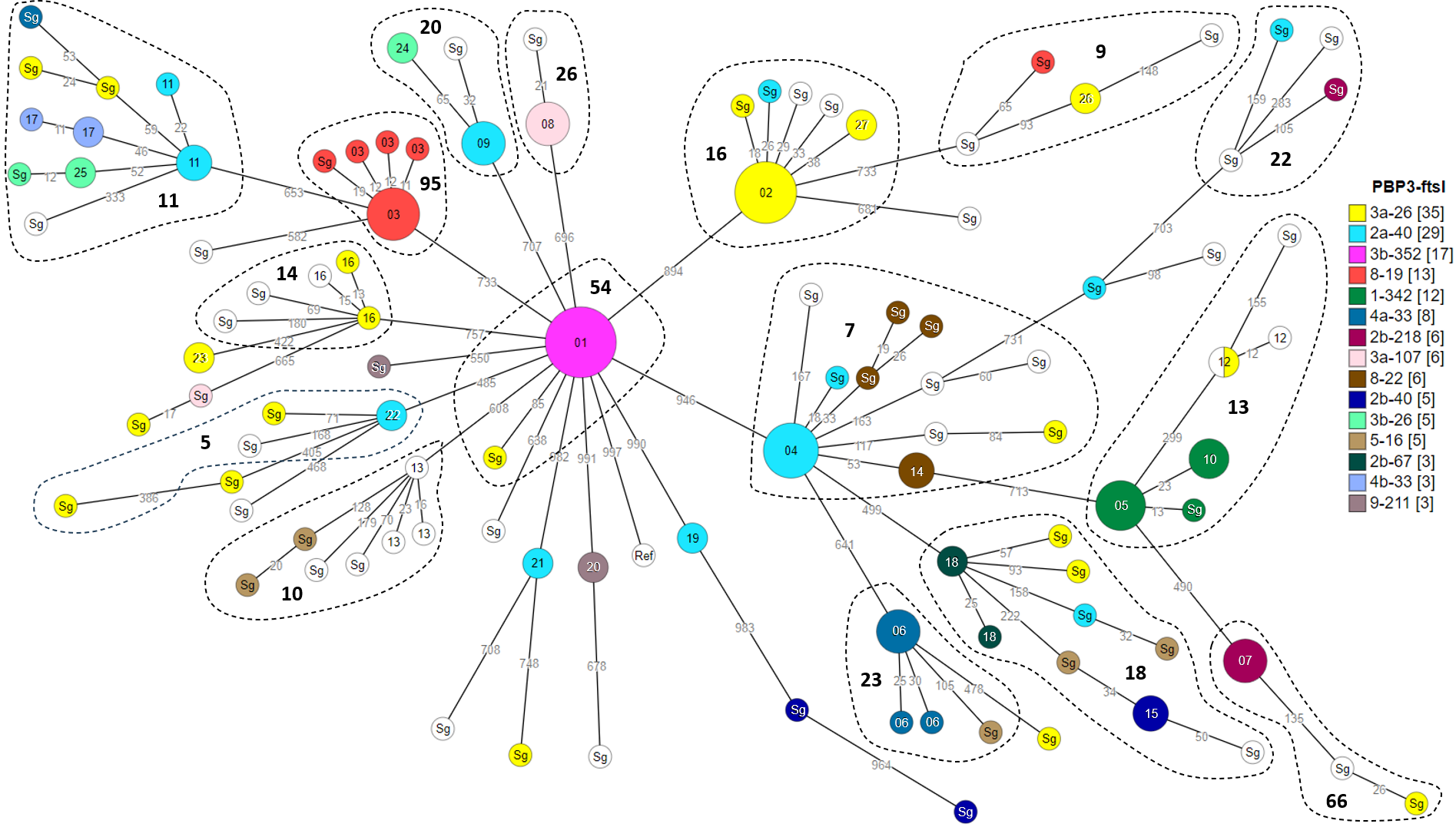


**Supplementary Figure S9.** Broth microdilution MIC distributions by rPBP3 stage and group for the included isolates (n=191) and eight beta-lactamase-stable beta-lactams. Box colors indicate rPBP3 stage (1, green; 2, orange; 3, red) (Figure 5). Group-specific median MICs (MIC50) are shown in the legends. Vertical lines indicate EUCAST breakpoints (v. 14.0, 2024). The meningitis breakpoint for meropenem is indicated by a dashed line.


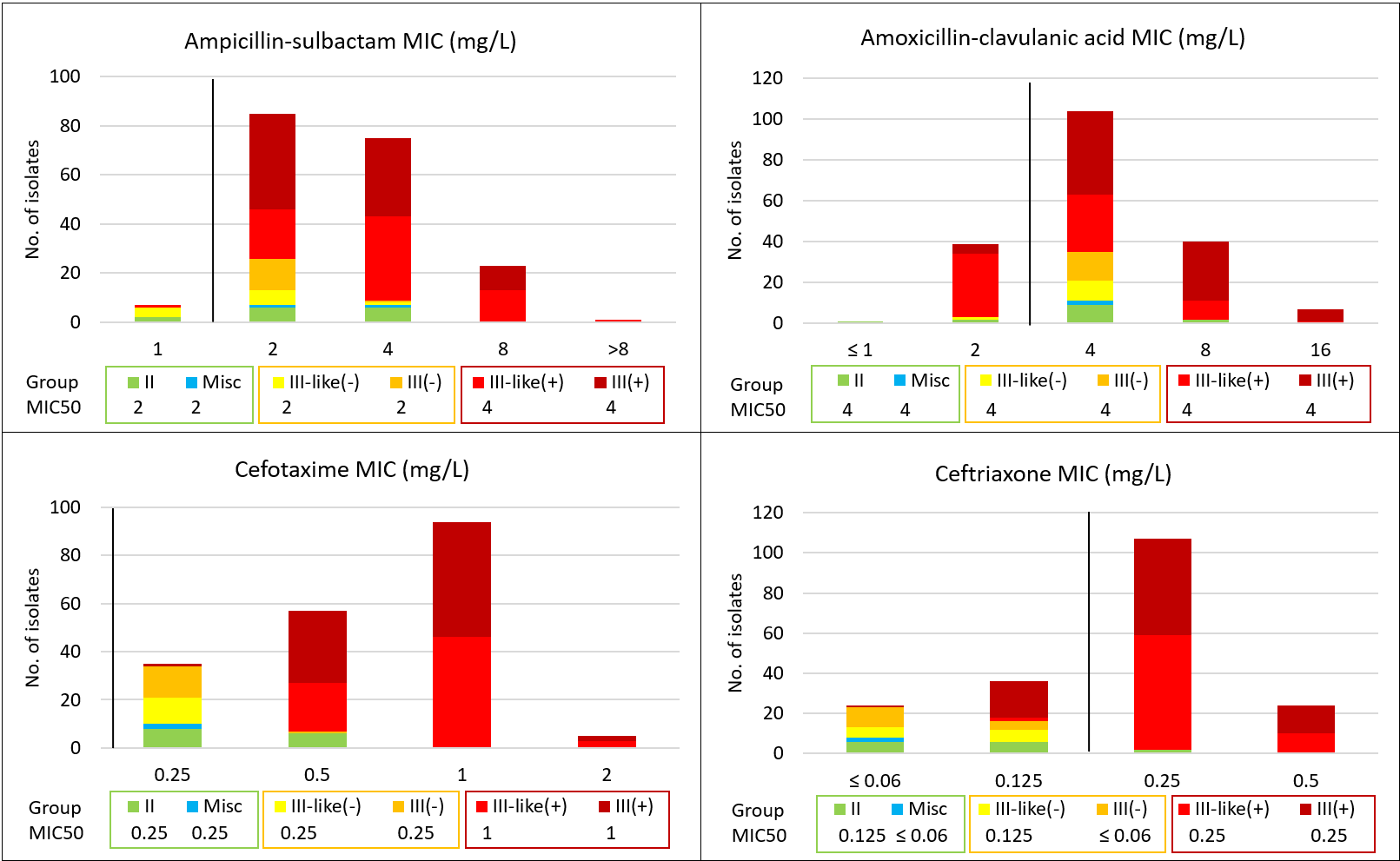


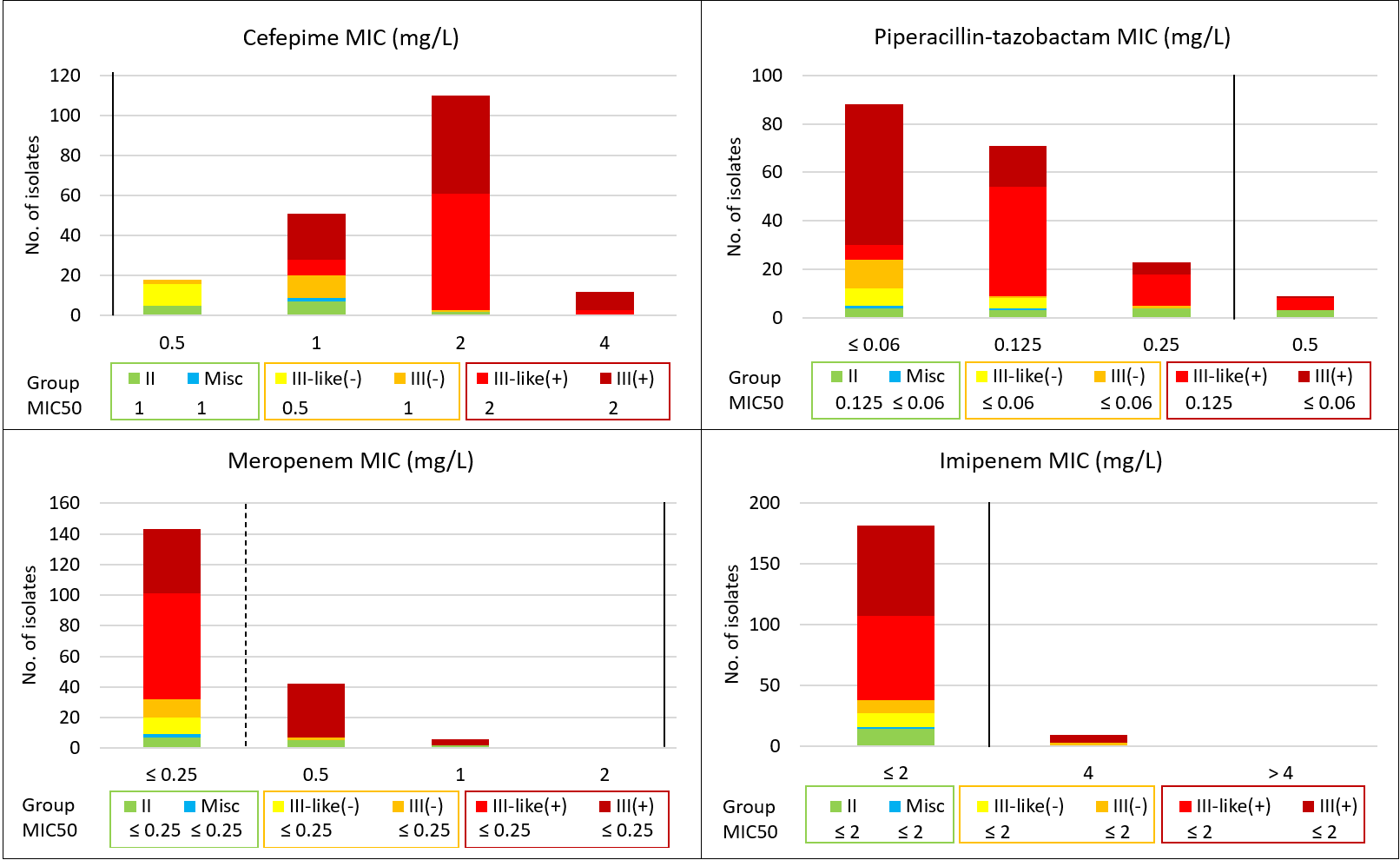


**Supplementary Figure S10.** Acquired resistance genes (n=13) and resistance gene profiles in the included isolates (n=191. Grey background shades indicate contigs. Contig orders are confirmed or tentative based on previously characterized integrative conjugative elements (ICE) in *H. influenzae* and *H. parainfluenzae*. In-depth characterization of cluster 18 (gene profile 2) and cluster 08 (gene profile 3) showed that all acquired resistance genes were carried on the ICEs Tn*6686* (Hegstad et al., 2020) and Tn*7100* (Johannessen et al., 2022), respectively.

**
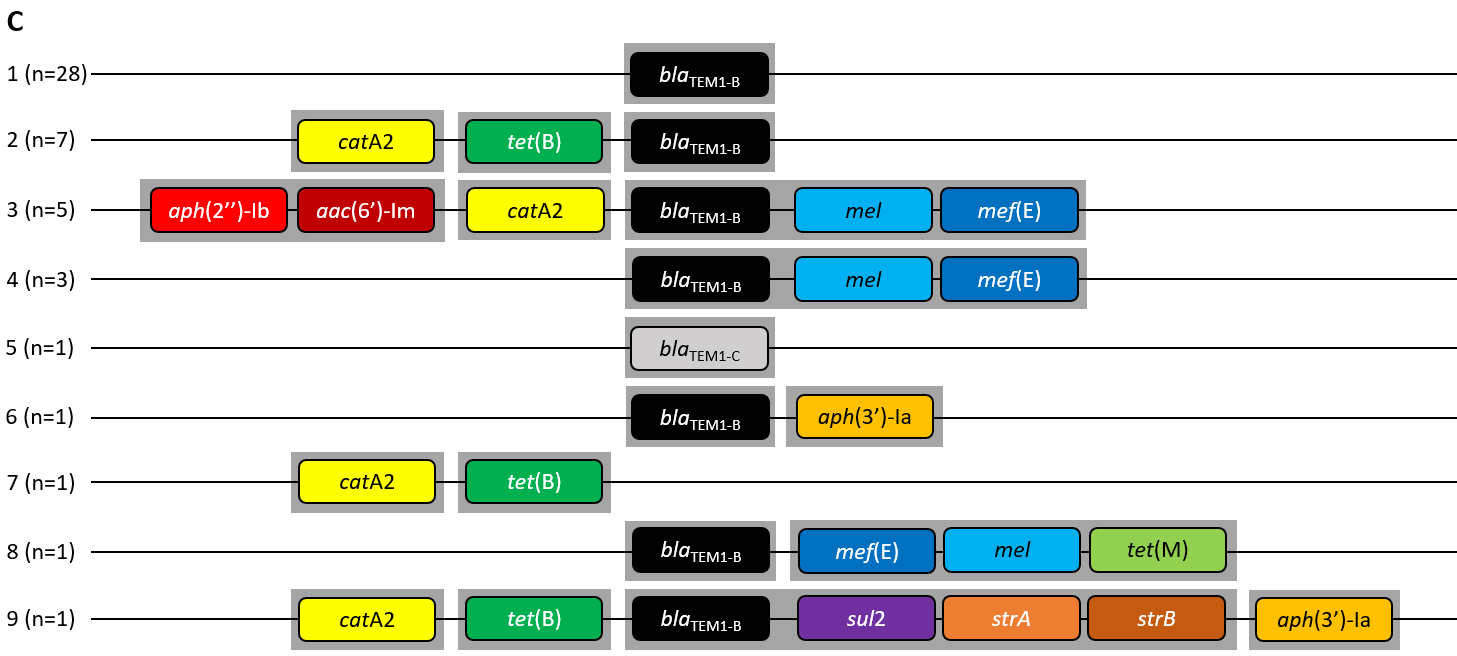
**

**Supplementary Figure S11.** Broth microdilution MIC distributions for the included isolates (n=191) and ten non-beta-lactams. Vertical lines indicate EUCAST breakpoints (v. 14.0, 2024) and epidemiological cut-off values (dashed lines). Columns, isolates with MIC > the R breakpoint and/or detected resistance determinants (acquired genes or chromosomal alterations); boxes, susceptible isolates (S or I) without known resistance determinants. Red and green columns indicate agents with and without clinical breakpoints, respectively. Dark and light colors indicate acquired genes and other determinants, respectively. Grey columns, silent gene or agent not tested. See Supplementary File for exact GyrA/ParC substitutions in quinolone-resistant isolates.


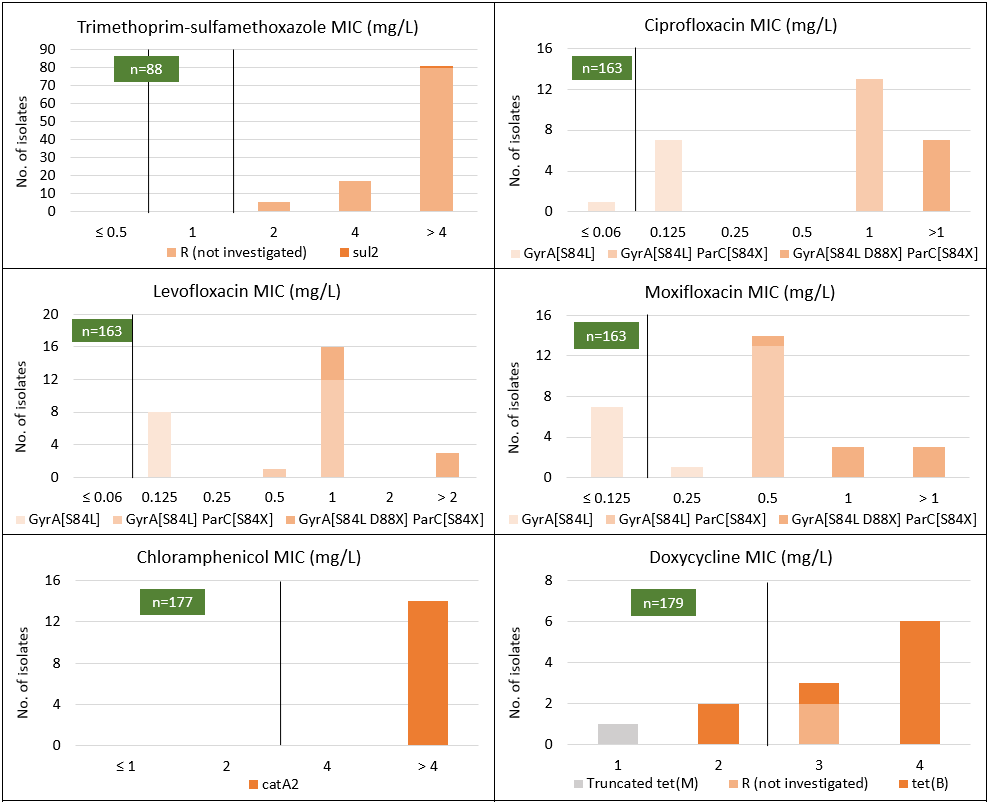


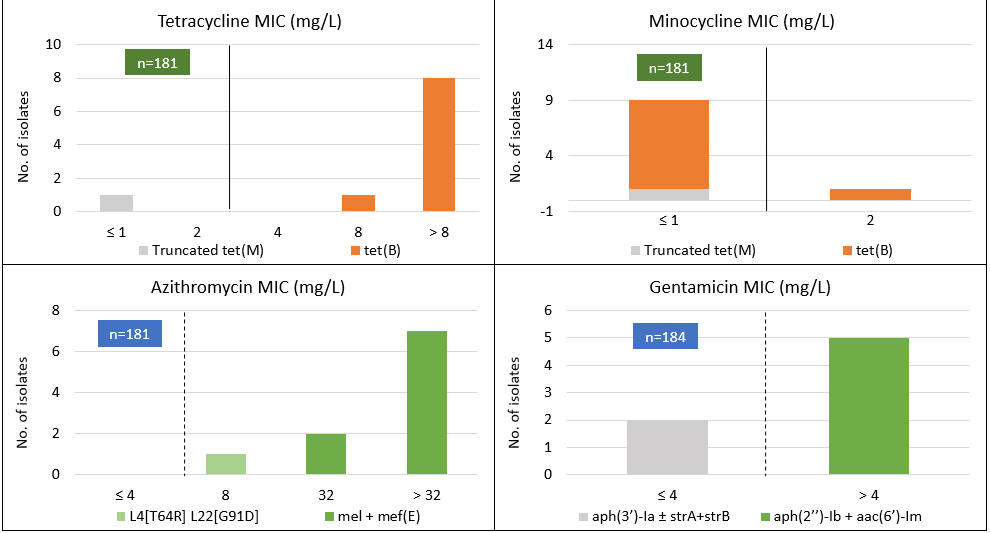


**Supplementary Figure S12.** Non-beta-lactam resistance rates for the included isolates (n=191) by rPBP3 stage (colored vertical bars). Overall resistance rates are shown as grey columns. Resistance rates according to Norwegian surveillance data (four agents) are shown as black bars (non-invasive, 2022). Asterisks indicate statistically significant differences between overall resistance rates and surveillance data (*p* < 0.001 for all agents). Agents with no detected resistance in the present study (rifampicin and tigecycline) are not shown.


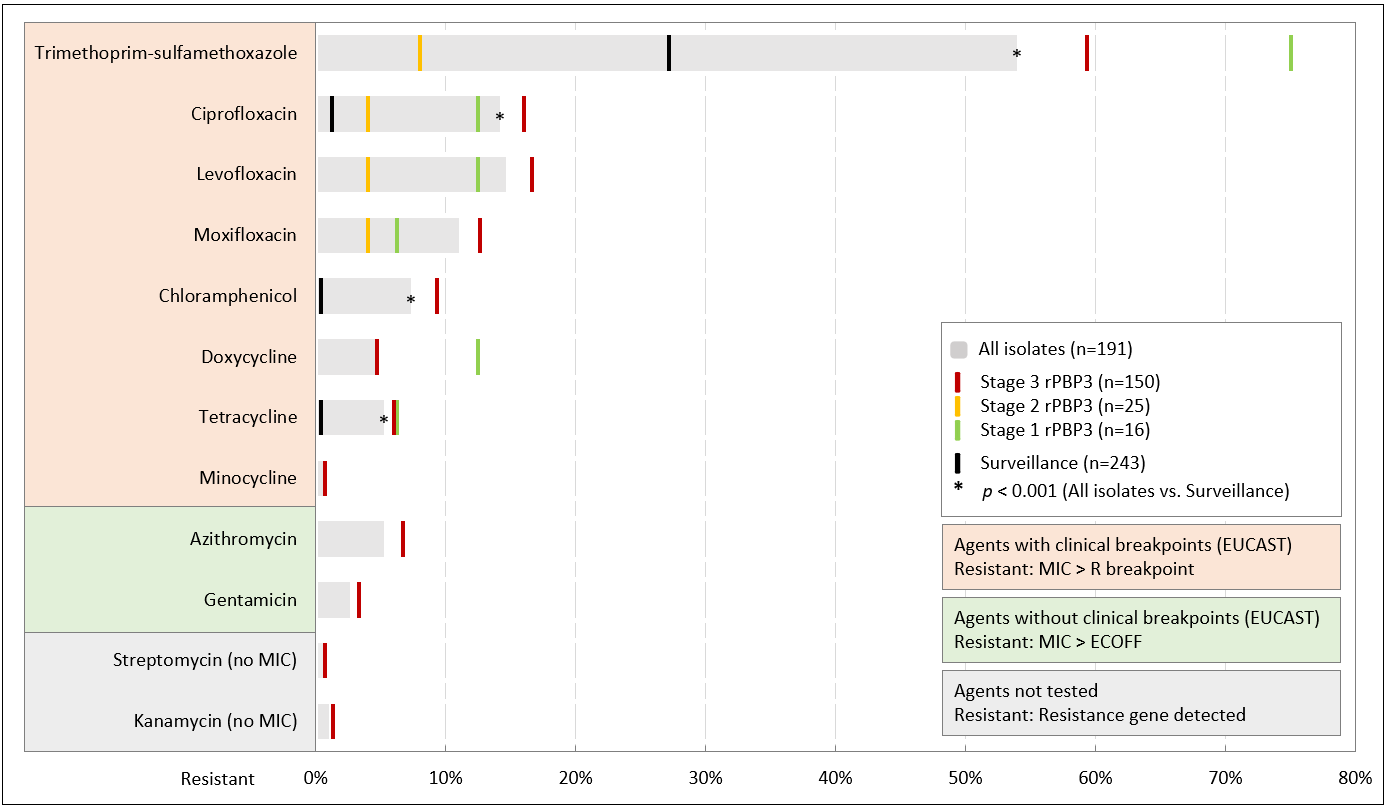


**Supplementary Figure S13.** Classification of multidrug resistance among the included isolates (n=191) by rPBP3 stage. The extent of multidrug resistance is expressed as the number of antimicrobial categories affected by resistance, i.e. MIC above the R breakpoint for at least one agent. Only agents with breakpoints for therapeutic use were counted (five beta-lactam and four non-beta-lactam categories) (Supplementary Table S1). MDR, multidrug-resistant; XDR, extensively drug-resistant.


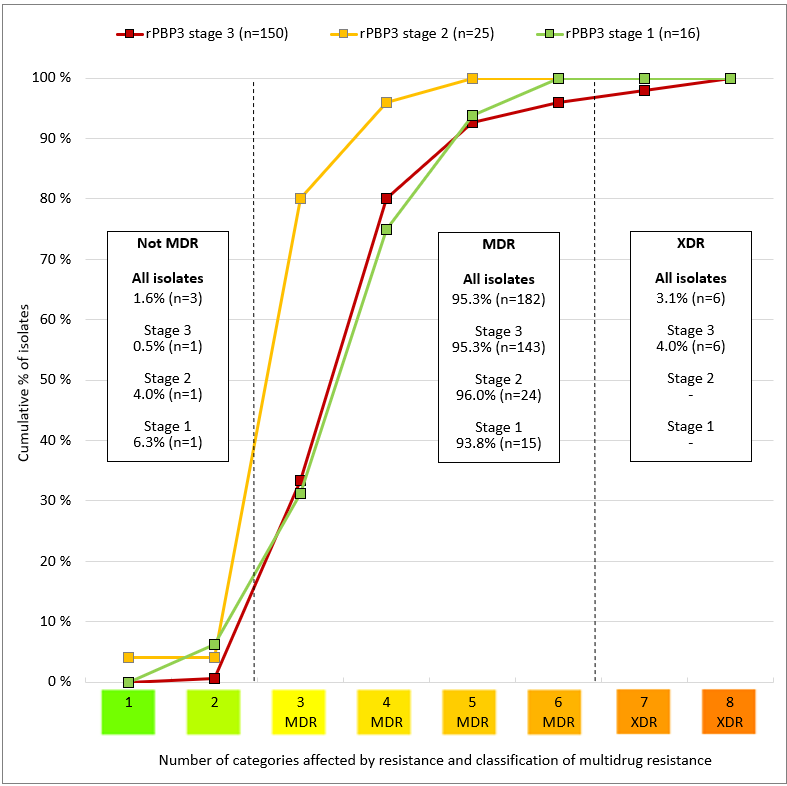


**Supplementary Figure S14.** Geographical distribution and molecular characteristics (LIN9, PopPUNK strain, ST [rPBP3 stage, PBP3-*ftsI* type]) of clusters with ≥ 4 members. Municipality of residence was used for geomapping and assignment to geographical region (Supplementary Figure S2). The bottom right tile shows distribution and characteristics of all included isolates (n=191). Cluster colors are the same as in Figure 4, Figure 6, Figure 8, and Supplementary Figure S7. Singletons have transparent nodes. The bar is 300 km.


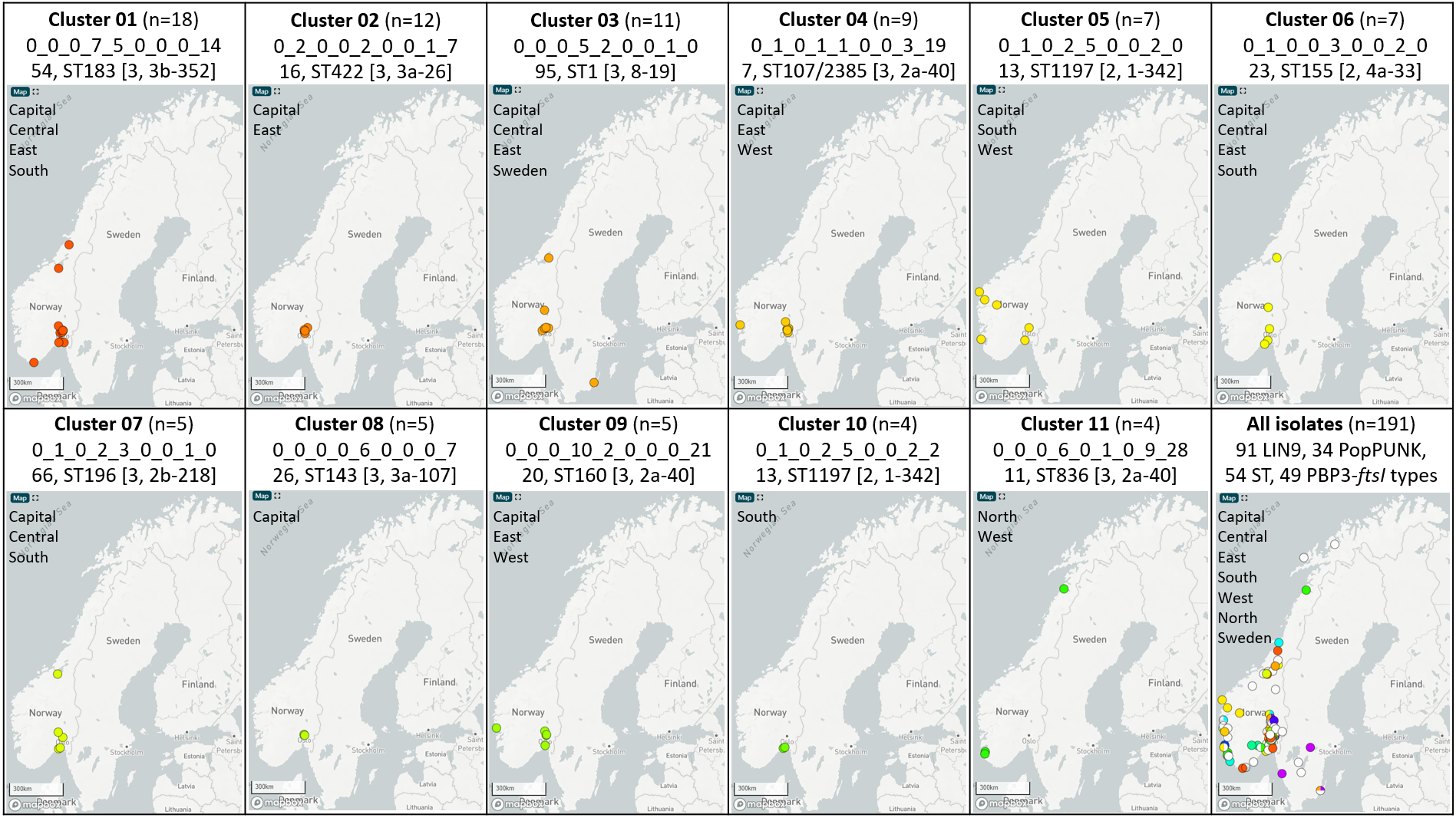

Supplement: Supplementary file 1 [file Table_1.docx]
